# Sex differences in amygdalohippocampal oscillations and neuronal activation in a rodent anxiety model and in response to infralimbic deep brain stimulation

**DOI:** 10.3389/fnbeh.2023.1122163

**Published:** 2023-02-23

**Authors:** Hanna Vila-Merkle, Alicia González-Martínez, Rut Campos-Jiménez, Joana Martínez-Ricós, Vicent Teruel-Martí, Ana Lloret, Arantxa Blasco-Serra, Ana Cervera-Ferri

**Affiliations:** ^1^Neuronal Circuits Laboratory, Department of Human Anatomy and Embryology, Faculty of Medicine, University of Valencia, Valencia, Spain; ^2^Department of Physiology, Faculty of Medicine, Health Research Institute INCLIVA, CIBERFES, University of Valencia, Valencia, Spain; ^3^Study Group for the Anatomical Substrate of Pain and Analgesia (GESADA) Laboratory, Department of Human Anatomy and Embryology, Faculty of Medicine and Odontology, University of Valencia, Valencia, Spain

**Keywords:** anxiety, depression, deep brain stimulation, hippocampus, amygdala, brain oscillations, sex differences

## Abstract

**Introduction:**

Depression and anxiety are highly comorbid mental disorders with marked sex differences. Both disorders show altered activity in the amygdala, hippocampus, and prefrontal cortex. Infralimbic deep brain stimulation (DBS-IL) has anxiolytic and antidepressant effects, but the underlying mechanisms remain unclear. We aimed to contribute to understanding sex differences in the neurobiology of these disorders.

**Methods:**

In male and female rats, we recorded neural oscillations along the dorsoventral axis of the hippocampus and the amygdala in response to an anxiogenic drug, FG-7142. Following this, we applied DBS-IL.

**Results:**

Surprisingly, in females, the anxiogenic drug failed to induce most of the changes observed in males. We found sex differences in slow, delta, theta, and beta oscillations, and the amygdalo-hippocampal communication in response to FG-7142, with modest changes in females. Females had a more prominent basal gamma, and the drug altered this band only in males. We also analyzed c-Fos expression in both sexes in stress-related structures in response to FG-7142, DBS-IL, and combined interventions. With the anxiogenic drug, females showed reduced expression in the nucleus incertus, amygdala, septohippocampal network, and neocortical levels. In both experiments, the DBS-IL reversed FG-7142-induced effects, with a more substantial effect in males than females.

**Discussion:**

Here, we show a reduced response in female rats which contrasts with the higher prevalence of anxiety in women but is consistent with other studies in rodents. Our results open compelling questions about sex differences in the neurobiology of anxiety and depression and their study in animal models.

## 1. Introduction

Depression and anxiety are the most common mental health problems, frequently appearing as comorbid illnesses (Pollack, 2005; Hranov, 2007; Campbell-Sills et al., 2012; Thibaut, 2017; Choi et al., 2020; Meuret et al., 2020). This phenomenon is of particular relevance since comorbid cases have a worse clinical outcome than each illness alone, with increased threat sensitivity and hyperarousal (Ironside et al., 2023), and a greater deterioration of the quality of life of individuals (Zhou et al., 2017; Saha et al., 2021). In addition, although the depressive disorder is the strongest predictor of suicidal behavior, comorbidity with anxiety increases the possibility markedly, up to 30%, of suicidal plans or attempts (Nock et al., 2010, 2013). However, it is still in discussion whether both disorders represent a common construct. Thus, research on the underlying altered brain networks and neurocomputation can help understanding their neurobiology and advance toward a personalized psychiatry (Chen, 2022).

One of the most consistent and remarkable findings in anxiety and depression is the existence of sex differences (Holden, 2005; Maeng and Milad, 2015; Jalnapurkar et al., 2018) in prevalence rates, symptomatology or disease course (Riecher-Rössler, 2017). The prevalence of depression and anxiety is consistently overrepresented in women than in men. According to the WHO, the prevalence of depression corresponds to 5.1% in females compared to 3.6% for males, and the prevalence of anxiety disorders is 4.6% in women compared to 2.6% in men (World Health Organization, 2019). Nevertheless, the most notable differences correspond to the comorbid cases of anxiety and depression, where women’s prevalence doubles that of men (McLean et al., 2011). In addition, women tend to have more symptoms in both disorders (Simonds and Whiffen, 2003; Schuch et al., 2014). Studies also report a more significant functional impairment and a higher probability of chronification and recurrence in both disorders in females (Marcus et al., 2008; Twenge et al., 2019; Höglund et al., 2020). Furthermore, some studies have shown that biological sex is an influential factor in antidepressant and anxiolytic pharmacological efficacy (Jalnapurkar et al., 2018; Eid et al., 2019). Also, in the last years, the study of sex differences in animal models has grown, leading a better understanding of the biological basis of mental disorders, but sometimes offering conflictive data for translating preclinical to clinical research (Tucker et al., 2022). However, sex as a biological variable remains underrepresented in this research field. This lack of research is more than surprising since the results of these findings could provide critical information for a better understanding of the etiological and pathogenic mechanisms of disorders which would probably lead to better therapeutic approaches (McCarthy et al., 2012). In this paper, we have used as a pharmacological anxiety model the drug FG-7142 (*beta*-*carboline*-*3*-*carboxylic acid ethyl ester methyl amide*). This compound has proven long-lasting anxiogenic capabilities (Skolnick et al., 1984; Thiébot et al., 1988) not only in animal models (Ninan et al., 1982; Ongini et al., 1983; Pellow and File, 1986; Lawther et al., 2015) but also in humans (Dorow, 1987; Horowski, 2020).

Despite the high relevance of depression and anxiety, current knowledge of the precise mechanisms underlying these pathologies are still poor compared to that of other chronic and debilitating diseases (Nestler and Hyman, 2010). At the cellular level, both pathologies share altered pathways, including mitochondrial impairment, which leads to reduced resilience under continuous stress (Tanaka et al., 2022). Between other alterations, depression and anxiety exhibit unbalanced neurotransmitters, impaired neuroplasticity, and abnormal neural circuitry (Pittenger and Duman, 2008; Liu et al., 2018). Identifying altered brain structures or circuits allows causal relationships with the disorders’ symptoms (Williams, 2016). In depression and anxiety, abnormal activity patterns arise in the amygdala-hippocampal-prefrontal network between other circuits. This network includes key structures in emotional and cognitive processing (McNaughton, 1997; Vanderlind et al., 2020).

Emotional processing largely depends on the activity of the amygdala and cortical areas located in the prefrontal regions (Etkin et al., 2015). Both anxiety and depression share a high negative hyperemotionality and an inability to regulate states and behaviors related to emotions and sensations (Elhamiasl et al., 2023). Neuroimaging studies have shown hyperactivation of the amygdala in response to threatening stimuli and negative emotional significance in depressed patients (Suslow et al., 2010; Victor et al., 2010). These abnormalities have also been observed in patients who, despite being under treatment, did not show any symptomatic remission (Drevets, 2001; Thomas et al., 2001; Suslow et al., 2010), in contrast with those who achieved an adequate response to the treatment (Hamilton et al., 2008).

In addition to emotional-related symptoms, depression and anxiety include cognitive alterations like memory dysfunction (primarily episodic and working memory), attentional biases, executive functions deficits, and cognitive and metacognitive vulnerabilities (Ferreri et al., 2011; Kircanski et al., 2012). While the management, integration, and recovery of memory depend on the hippocampus (Squire, 1992; Wallenstein et al., 1998), the executive, attentional functions and goal-directed behaviors have been related to the prefrontal cortex (PFC) (Koechlin et al., 1999; Miller et al., 2002). PFC dysfunction affects depressed patients’ memory and emotional learning capacity. Specifically, smaller volume and altered activity patterns of the ventromedial region of PFC (vmPFC) have been observed in patients with depressive disorder, contributing to fear extinction deficits (Battaglia et al., 2022).

The hippocampus contributes to emotional and cognitive processing, with a differentiated role along the longitudinal axis of the hippocampus (Strange et al., 2014). The dorsal hippocampus (HPCd) is key for learning and episodic memory (Moser et al., 1993; Moser and Moser, 1998) and spatial mapping (O’Keefe and Nadel, 1978). In contrast, the ventral hippocampus (HPCv) is involved in emotional memory, stress processing (Bannerman et al., 2004; Fanselow and Dong, 2010), and anxiety (Bannerman et al., 2004; Adhikari et al., 2010). However, its role is still unclear since discrete neuronal populations in the HPCv either promote or suppress anxiety through different projection targets (Parfitt et al., 2017) with sexual differences in mice (Wang et al., 2019). To date, little is known about the role of HPCi. However, this region has been proposed as a spatial, cognitive, and emotional integrator (Bast et al., 2009; Jin and Lee, 2021). Reduced hippocampal volumes have been observed in depression (Campbell and Macqueen, 2004; Videbech and Ravnkilde, 2004), correlating with greater severity and duration of the disease (McKinnon et al., 2009) and reversed by antidepressant drug treatment (Sheline et al., 2003). Abnormalities in the hippocampal microstructure also appear in comorbid anxiety with depression, associated with an exacerbated processing of threats (Cha et al., 2016). In turn, hippocampal damage or atrophy leads to poor stress processing, given its control over the hypothalamic-pituitary axis (Sapolsky, 2001).

Deep brain stimulation (DBS) consists of the implantation of electrodes in nuclei or bundles of brain fibers to supply currents that alter neural function therapeutically. Since Mayberg et al.’s (2005) seminal work, DBS has been applied in the anterior cingulate cortex (ACC) as a surgical approach in cases of treatment-resistant depression (Schlaepfer and Bewernick, 2013; Zhou et al., 2018; Crowell et al., 2019) and other targets like the median forebrain bundle (Rymaszewska et al., 2023). Recent meta-analyses corroborate its efficacy despite requiring more clinical and preclinical studies that facilitate a better understanding of its mechanisms of action (Khairuddin et al., 2020; Zhang et al., 2021). DBS has also been applied in the closest correlate in rodents to the ACC, the infralimbic cortex (DBS-IL), with antidepressant and anxiolytic effects in different behavioral tests (Gersner et al., 2010; Hamani et al., 2010; Hamani and Temel, 2012; Schmuckermair et al., 2013; Veerakumar et al., 2014; Jiménez-Sánchez et al., 2016; Torres-Sanchez et al., 2018).

Brain oscillations contribute to encoding, integration, retrieval, and transmission of neural information (Sejnowski and Paulsen, 2006). Therefore, aberrant brain oscillations would reflect altered neuronal dynamics or could lead to the development of a neurological or mental disorder (Başar, 2013), including depression and anxiety (Uhlhaas and Singer, 2012). In these pathologies, preclinical studies and clinical trials have focused mainly on the alpha, theta, and gamma waves (Knyazev et al., 2004, 2005; Smart et al., 2015; Fitzgerald and Watson, 2018; Fernández-Palleiro et al., 2020; Ippolito et al., 2022). There is also evidence of the alteration of slow waves, beta, and delta in depression and anxiety (Başar et al., 2001; Li et al., 2017; Muir et al., 2020).

We analyzed the neural oscillations of this network under the effects of the anxiogenic drug FG-7142 in male rats (Vila-Merkle et al., 2021). In that study, a characteristic communication pattern was observed. Slow waves disappeared, and a sustained theta emerged in hippocampal regions, with delta and beta oscillations in the HPCv and amygdala. This pattern was reversed by DBS of the infralimbic prefrontal cortex (DBS-IL).

However, this previous study was conducted solely in males. In the present paper, we compare the communication in the amygdalohippocampal network in male and female rats to contrast whether our prior observations are also valid in females. Most studies in animal models are conducted in male rodents, so research on females is also needed to understand how neural networks operate. We expected to find an increased response to the anxiogenic drug. In contrast to our expectations, when trying to reproduce these experiments in female rats, the results were remarkably different, with minor activity in the network. The present study also includes the analysis in both sexes of neuronal activation by c-Fos expression in several brain structures involved in stress, anxiety, depression, or fear in response to FG-7142, DBS-IL alone, and the combination of both. With this study, we aim to contribute to understanding the neurobiology of anxiety and depression and their comorbidity, highlighting the sexual differences in these animal models.

## 2. Materials and methods

### 2.1. Subjects

A total of 60 adult Wistar rats (Charles River Company, Barcelona, Spain) were included in the electrophysiological study of the amygdalo-hippocampal network. Specifically, six male and six female rats were used for the preliminary study of FG-7142, and 24 male and 24 female rats were used to study the effect of DBS-IL in anxiety conditions. The study of c-fos expression under the effect of FG-7142, DBS-IL, and the combination of both experimental procedures included 40 adult Wistar rats (20 females and 20 males; Charles River Company, Barcelona, Spain). All animals used aged 3–6 months, weighing between 250 and 350 g.

All animals were housed in the Central Research Unit at the University of Valencia (Spain) under a 12-h light/dark cycle and a controlled and constant room temperature of 22 ± 2°C and humidity of 55 ± 10%, with *ad libitum* access to food and water. All the experimental protocols were followed according to the Animal Care Guidelines of the European Communities Council Directive (2010/63/E.U.) and approved by the Research Ethics and Animal Welfare Committee of the University of Valencia (A20200227130134, A20191121151842, and A1537174325669) and the Valencian Government (Generalitat Valenciana) before performing the experiments.

The data from male rats were used in a previous paper (Vila-Merkle et al., 2021). The surgical procedures, *in vivo* electrophysiological recordings, drug administration, stimulation procedure, electrophysiological data analysis, and histological verification of the recording sites are thoroughly described in the aforementioned publication. They are briefly described in the following sections to facilitate manuscript understanding.

### 2.2. Surgical procedures

All surgical procedures were performed under urethane (1.5 g/kg; Sigma-Aldrich/Merck, Barcelona, Spain) anesthesia. To record the local field potentials, custom-made Teflon-coated stainless steel recording electrodes (AM Systems, Sequim, WA, USA) were implanted, according to the stereotactic coordinates from Bregma (Paxinos and Watson, 2007), in the dorsal hippocampus (HPCd) (AP −3.4 mm; L 2.5 mm; DV 2.4 mm), intermediate hippocampus (HPCi) (AP −5.8 mm; L 5.8 mm; DV 5 mm), ventral hippocampus (HPCv) (AP −4.7 mm; L 5 mm; DV 8.7 mm), and basolateral amygdala (BLA) (AP −2.3 mm; L 5 mm; DV 8.5 mm). For DBS, we used in-house custom-made bipolar twisted electrodes made of Teflon-coated stainless-steel wire, with 1 mm between both tips. Stimulating electrodes were bilaterally implanted into the infralimbic (IL) region of the medial prefrontal cortex (coordinates: AP +3.2 mm; L 0.5 mm; DV 5.4 mm).

**FIGURE 1:**
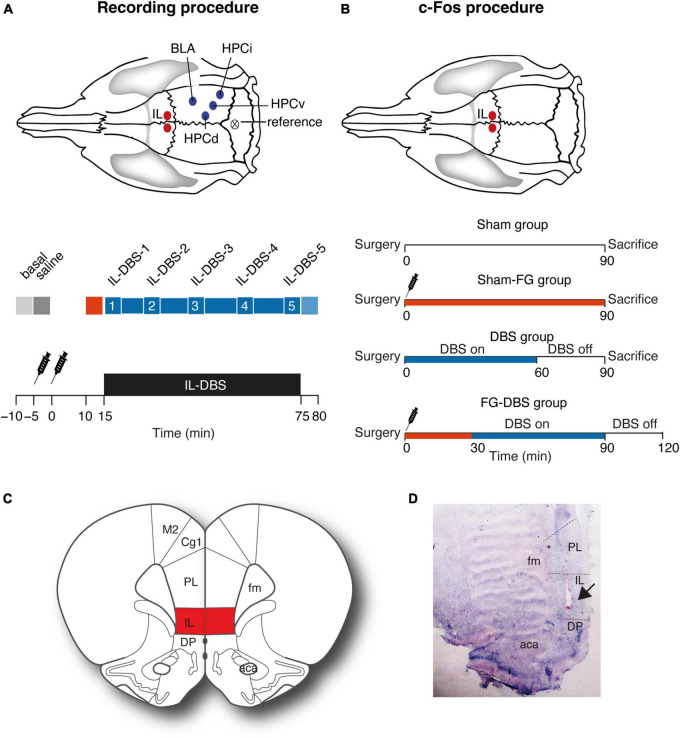
Experimental setup. **(A)** Electrophysiological recording experiment. Recording and stimulating regions and electrophysiological recording protocol with periods selected for statistical analysis. Red: Period with FG-7142 effect. Blue: 1 h of DBS application. 1–5 indicate different time periods during the DBS stimulation. **(B)** c-Fos experiment. Stimulating region and protocol for c-Fos analysis. The syringe indicates FG-7142 administration. **(C)** Location of the infralimbic region. **(D)** Histological verification of the electrode placement (arrow). Please note that the stimulation was bilateral, although the image only shows one hemisphere. Aca, anterior commissure; BLA, basolateral amygdala; CG, cingulate cortex; DP, dorsal peduncular cortex; HPCd, dorsal hippocampus; HPCi, intermediate hippocampus; IL, infralimbic cortex; HPCv, ventral hippocampus; M1, motor cortex; PL, prelimbic cortex.

The surgical procedures used in the study of the electrophysiological activity are the same as for the analysis of c-Fos expression. However, in the case of the immunoreactivity study, only the DBS stimulation electrode in IL was implanted to preserve an optimal state in the other regions of interest. Figure 1 illustrates the experimental setup and the stimulation sites.

### 2.3. Drug administration

To achieve an anxiogenic state, the animals received an intraperitoneal injection of FG-7142 (beta-Carboline-3 carboxylic acid-N-methyl amide; Sigma Aldrich, St. Louis, MO, USA), an inverse partial agonist of benzodiazepines, in a single dose of 7.5 mg/kg, dissolved in one-Cyclodextrin solution (Sigma Aldrich, St. Louis, MO, USA) dissolved in a sterile saline solution acidified to 1 M hydrochloric acid (HCl) pH5 to a final volume of 2 ml/kg following the procedures of Lawther et al. (2015).

### 2.4. Deep brain stimulation parameters

The DBS-IL was performed bilaterally in both experiments using an electrical pulse generator. The stimulation device was manufactured by the Digital Signal Processing Group of the University of Valencia in collaboration with the Neuronal Circuits Laboratory.

We selected the DBS paradigm described by Hamani et al. (2010): continuous electrical pulse trains at 130 Hz, 100 μA, and 90 ms. This stimulation pattern generates a charge density per phase (electric current × pulse width/mm^2^ of electrode surface) that approximates the paradigm used in clinical practice.

### 2.5. *In vivo* electrophysiological recordings

One hour after surgery, the recording session began. The experimental procedure involved a continuous recording under anesthesia in the following conditions: (1) 300 s as baseline (henceforth, “basal” epoch); (2) 300 s following the administration of saline solution (“saline” epoch) as control; (3) administration of the anxiogenic drug and, subsequently, recording for 15 min (“FG-7142” epoch); (4) 1 h of DBS-IL (bipolar stimulation at 130 Hz, 100 μA, and 80 μs) (“DBS” epoch); (5) 300 s in DBS-IL-off mode (“post-DBS” epoch). An additional group (six males and six females with implanted electrodes) only received saline and FG-7142 but were in DBS-off mode during all the recordings to visualize the time course of the response to the drug and the intervention alone (Figure 1A).

Raw signals were preamplified (model p511 AC Grass Preamplifier) and amplified (MPLI 4G21; Cibertec, Madrid, Spain), online bandpass filtered between 0.3 and 300 Hz, and the 50 Hz noise was removed (Hum Bug; Quest Scientific, North Vancouver, BC, Canada). Then the signals were digitized (CED Micro; Cambridge Electronics Design, Cambridge, UK) for offline analysis (1,000 Hz sampling frequency). The waveforms were recorded and monitored online using Spike 2 software (Cambridge Electronics Design).

### 2.6. Histological verification of the recording sites

After the recordings, animals were deeply anesthetized with sodium pentobarbital (100 mg/kg; Dolethal, Vetoquinol, Madrid, Spain) and transcardially perfused with heparinized saline solution (0.9%, pH 7), followed by 4% paraformaldehyde (Sigma-Aldrich, St. Louis, MO, USA) diluted in phosphate buffer (PB, 0.1 M, pH 7.6). Brains were removed and postfixed overnight in the same fixative and cryoprotected solution in 30% sucrose in PB (0.1 M, pH 7.6) at 4C until they sank. Coronal sections of 40 μm were obtained by a freezing microtome (Leica, Madrid, Spain), collected in the same solution, and stored for processing. Sections were then stained by the Giemsa technique to verify the placement of electrodes and the injection site subsequently. Attending to our aim, only those cases in which the electrode tips were clearly positioned within the boundaries were included in the study. Figure 1 illustrates the placement of the stimulating electrodes.

### 2.7. Electrophysiological data analysis

#### 2.7.1. Spectral analysis

The local field potentials recordings were based on the Fast Fourier Transform. To calculate power spectral density, we applied the Welch method in 5 s overlapped windows (50%) and a value for nfft parameter of 1,024 with the Signal Processing MATLAB Toolbox. The analyzed bands were slow oscillations (SW < 1.5 Hz), delta (1.5–2.5 Hz), low theta (2.5–5 Hz), high theta (5–12 Hz), beta (16–30 Hz), low gamma (30–60 Hz), mid gamma (60–90 Hz), and high gamma (90–120 Hz). Spectral power and relative power were calculated for each band in consecutive 60 s windows to visualize the time course of each band. Relative power was calculated as band power/power in the 0–250 Hz.

#### 2.7.2. Wavelet analysis

Given that theta oscillations play a suitable role in hippocampal processing and within limbic structures, we examined this oscillation in further detail by detecting epochs with predominant low theta activity (“theta segments”). To improve the spectral analysis, we used a continuous wavelet transform to enhance the analysis on the time-frequency domain.

Theta segments were determined as 2.5–5 Hz continuous oscillations constituting at least 30% of the total oscillatory activity for each time point and computed in consecutive 60 s windows. The analysis of theta segments allowed the quantification of the temporal ratio (proportion of time with predominant theta segments/s), the mean duration, and the number of segments for each window.

Wavelet coherograms were used for visualizing the coupling between channels. To analyze the synchrony between the recorded areas, we used the phase-locking value (PLV), defined as a measure of the stationarity of the phase differences in 10 s temporal windows. The index used here was the weighted phase lag index (WPLI), in which the contribution of the observed phase leads and lags is weighted by the magnitude of the imaginary component of the cross-spectrum. This index increases the specificity by reducing the contribution of noise sources or volume conduction and the statistical power to detect changes in phase synchronization.

#### 2.7.3. Cross-frequency coupling

Phase–amplitude coupling (PAC) was estimated in MATLAB *via* the modulation index (MI), as defined by Tort et al. (2010) and defined before. The MI was normalized to the 0–1 range and was assessed statistically significant when its value was greater than two standard deviations of the substitute mean MI, constructed by 200 random permutations of the amplitude distribution.

#### 2.7.4. Histological processing and cell counting

The study of neural activation was carried out in a total number of 47 brain regions in male and female rats according to the following experimental paradigm, all under urethane anesthesia: (1) sham group: animals that only underwent implantation surgery; (2) DBS group: animals receiving 1 h from DBS-IL; (3) sham-FG group: animals with FG-7142 administration and a DBS-IL electrode implanted but without performing the stimulation; and (4) FG-DBS group: animals receiving 1 h of DBS-IL and FG-7142 administration (Figure 1C).

90 min after the animals received the treatment, they were transcardially perfused with 500 mL of heparinized saline followed by 500 mL of 4% PFA. Brains were postfixed overnight in the same fixative and cryoprotected. Forty μm-thick coronal slices were obtained in a freezing microtome and collected in six parallel series. Selected slices were washed with Tris-buffered saline (TBS, 0.05M, pH 7.6) and treated for 30 min in TBS with 1% Triton X-100 (TBS-T; PanReac AppliChem) and 30% H_2_O_2_. Brain sections were then preincubated for 120 min at room temperature in a TBS-T blocking solution with 2% bovine serum albumin (BSA) and 3% normal goat serum (NGS, Normal Goat Serum, Thermo Fisher, Waltham, MA, USA). Afterward, sections were incubated in blocking solution (2% BSA, 3% NGS and 1% Tx- 100 in TBS) with recombinant monoclonal Rabbit anti c-Fos (1:1000; Synaptic Systems, Göttingen, Germany) for 24 h at 4°C. After washing, slices were incubated in a solution with TBS-T and the Goat/anti-Rabbit biotinylated IgG (1:200; Thermo Fisher, Waltham, MA, USA) for 120 min at room temperature.

Finally, sections were then incubated in the avidin-biotin complex (PK4000 Vectastain ABC Kit c, Abcam), according to the commercial specifications, and counterstained in a solution with TRIS, 0.04% DAB (3,3′-Diaminobenzidine) and 30% H_2_O_2_, in a period of between 10 and 30 min until the desired color saturation is achieved.

Stained sections were mounted onto gelatinized glass slides and covered with DPX (Merck, Barcelona, Spain). Sections were selected with an optical microscope (Axioscope A1, Zeiss Microscopy, Munich, Germany) and subsequently photographed using a sequential scanning mode with a high-resolution refrigerated camera attached to it (Axiocam MRC, Carl Zeiss). To capture the images, Zen software (Zeiss Microscopy, Munich, Germany) was used. Quantitative data were obtained by automated image analysis with a self-written FIJI macro (2.1.0 153i, ImageJ, Bethesda, Maryland, USA).

#### 2.7.5. Statistical analysis

Statistical analyses and graphical representation were performed using R Studio software (v.1.4; PBC, Boston, MA, USA). Non-parametric tests were selected for the implementation of the statistical study analysis after verifying through the Shapiro–Wilks test that the sample did not assume a normal distribution. For the study of local field activity, the Friedman chi-square test was applied with the Conover test as *post hoc*. The immunoreactive c-fos expression statistical analysis was performed using the Kruskal–Wallis H test. The study of sex differences was carried out using the Mann–Whitney test with the Bonferroni *post hoc*. A significance level of *p*-value < 0.05 was considered to determine the existence of statistically significant differences. Statistical data are expressed as mean ± standard error.

## 3. Results

### 3.1. Comparison of the oscillatory profile between male and female rats with FG-7142 administration

**FIGURE 2:**
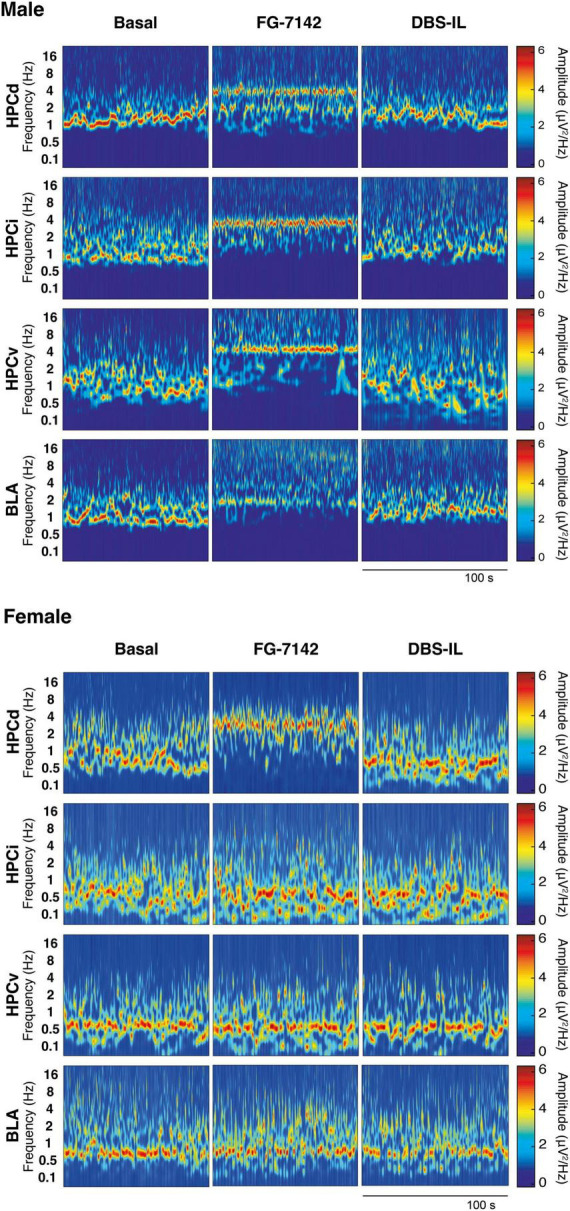
Representative wavelet spectrograms in the HPCd, HPCv, and BLA in basal conditions and after FG-7142 and DBS-IL interventions in male and in female rats. Observe the frequency increase induced by FG-7142 in all channels and the reversal of the oscillatory profile to slow waves by DBS-IL in males. In females, a reaction to FG-7142 is only observed in HPCd. BLA, basolateral amygdala; HPCd, dorsal hippocampus; HPCi, intermediate hippocampus; HPCv, ventral hippocampus.

To characterize the oscillatory activity and the duration of the reverse benzodiazepine FG-7142 (7.5 mg/kg), local field potential (LFPs) recordings were performed in the amygdala-hippocampal circuit in anesthetized male and female Wistar rats (*n* = 6 in each group). Figure 2 shows the oscillatory profile of FG-7142 in a representative male and female case, respectively.

As expected, in both groups, urethane anesthesia was characterized by the predominant presence of a low-voltage oscillatory pattern in the slow waves range (<1.5 Hz) and delta activity (1.5–2.5 Hz). Simultaneously, the basal period presented spontaneous alternating scattered, and short theta activity. Saline administration unaltered this pattern, while FG-7142 (i.p.) induced differences between both sexes. Males characteristic oscillatory profile shifted to faster frequencies, recognizable in all channels, lasting 2 h after drug administration. In females, this effect could be consistently observed only in the HPCd. The remaining channels did not display an apparent increase in frequency, or their duration was much shorter.

### 3.2. Peak frequency

**FIGURE 3:**
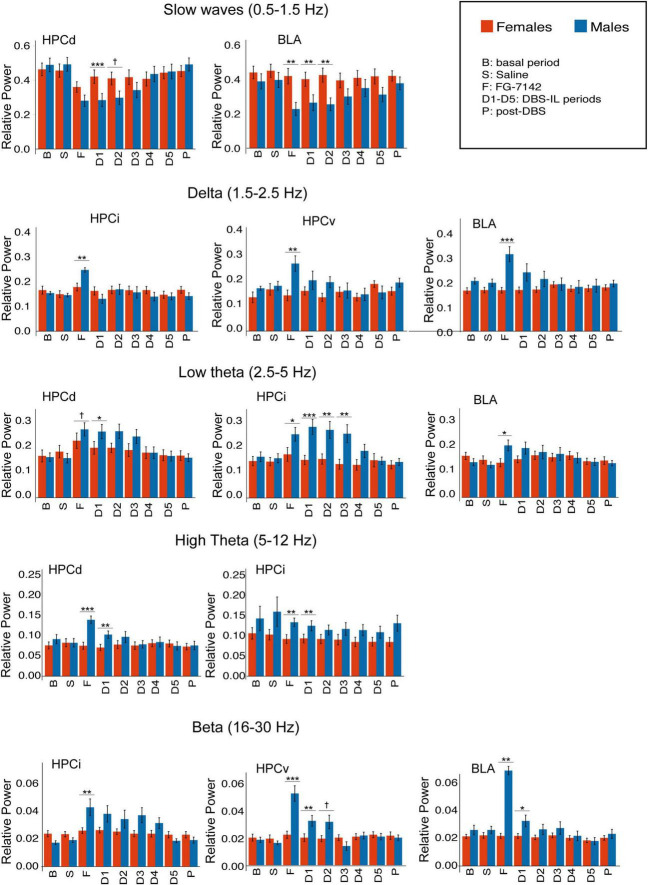
Sex differences in relative power, calculated by spectral decomposition. Only regions with statistical differences are shown. FG-7142 decreases slow waves in HPCd in both sexes, but males had a marked decrease in BLA. FG-7142 only increased delta and beta activity in HPCi, HPCv, and especially BLA in males. The drug also raised the low theta in HPCd in both sexes but had a higher effect in males. High theta was increased only in HPCd and HPCi in males. The DBS restored the alterations in all cases, with the most marked effect between sexes in the first periods (D1-D3). Asterisks denote the degree of statistical significance in the pairwise comparisons between sexes (***p < 0.001, **p < 0.01, *p < 0.05). The specific values corresponding to each period can be consulted in Supplementary Table 1. B: basal; S: saline; F: FG-7142; D1-D5: DBS-IL periods 1–5; P: post-DBS. BLA, basolateral amygdala; DBS-IL, deep brain stimulation of the infralimbic cortex; HPCd, dorsal hippocampus; HPCi, intermediate hippocampus; HPCv, ventral hippocampus. ^†^Denotes: *p* < 0.08.

The peak frequency was analyzed to characterize precisely the changes induced by FG-7142 and the effect of DBS-IL on the oscillatory profile (Figure 3). For this aim, the Fast-Fourier Transform (FFT) was applied in consecutive windows of 60 s throughout all recordings. This allowed us to study the time course of the peak frequency and the extraction of representative windows averaged over 300 s duration for each period to be analyzed statistically. Table 1 summarizes the statistical results.

**TABLE 1:** Peak Frequency in both sexes.

Sex	Period	dHPC	HPCi	HPCv	BLA
Male	Basal	1.063±0.039	1.084±0.042	0.979±0.051	1.105±0.045
Female	Basal	1.040±0.048	1.024±0.045	1.057±0.086	1.132±0.047
Male	Saline	1.042±0.046	1.041±0.063	1.048±0.069	1.084±0.042
Female	Saline	1.060±0.061	1.069±0.056	1.085±0.091	1.190±0.050
Male	FG-7142	**3.355**±0.128***	**3.574**±0.100***	**3.629**±0.244***	**3.202**±0.083***
Female	FG-7142	**1.223**±0.068***	**1.214**±0.072***	**1.182**±0.119***	**1.187**±0.049***
Male	DBS1	**3.047**±0.087***	**3.356**±0.056***	**3.330**±0.087***	**3.526**±0.061***
Female	DBS1	**1.281**±0.096***	**1.190**±0.090***	**1.183**±0.094***	**1.107**±0.063***
Male	DBS2	**2.380**±0.098***	**2.292**±0.081***	**1.705**±0.085***	**1.653**±0.109***
Female	DBS2	**1.133**±0.071***	**1.242**±0.089***	**1.040**±0.096***	**1.121**±0.058***
Male	DBS3	1.251±0.051	1.421±0.101	**1.426**±0.061**	1.359±0.101
Female	DBS3	1.174±0.089	1.209±0.077	**1.099**±0.055**	1.210±0.060
Male	DBS4	1.147±0.058	1.230±0.051	**1.253**±0.098*	**1.398**±0.046***
Female	DBS4	1.265±0.086	1.136±0.081	**0.976**±0.066*	**1.071**±0.060***
Male	DBS5	1.083±0.060	1.147±0.049	1.048±0.070	1.147±0.058
Female	DBS5	1.131±0.077	1.165±0.082	1.264±0.150	1.153±0.072
Male	POST	1.063±0.049	1.020±0.060	1.048±0.069	1.063±0.049
Female	POST	1.054±0.039	1.072±0.050	1.039±0.035	1.083±0.035

Mean ± standard error. Bold: statistical significance between sexes; asterisks denote statistical significance between states ***p < 0.001, **p < 0.01, *p < 0.05. BLA, basolateral amygdala; HPCd, dorsal hippocampus; HPCi, intermediate hippocampus; HPCv, ventral hippocampus.

In males, the FG-7142 induced a marked and significant reduction of slow wave activity (<1.5 Hz) in all regions, causing a regular oscillatory pattern with an increase in the peak frequency, which appeared robustly in the low theta range, between 3 and 3.5 Hz. This effect was progressively reversed until returning to baseline states approximately 45 min after its onset [see Vila-Merkle et al. (2021) for details]. The administration of FG-7142 in females slightly increased the frequency only in the HPCd and HPCi, without reaching the low theta range since the peak frequency was established in the band of slow waves around 1.3 Hz. DBS-IL restored the peak frequency in these regions approximately 45 min after its application, returning to the basal frequencies. However, HPCv and BLA did not experience any change relative to the peak frequency in any of the analyzed periods.

Comparative analysis of the results confirmed the existence of sex differences related to the frequency peak, both in response to FG-7142 and in the first stages of the application of DBS-IL. Specifically, all the recorded areas showed significant differences (*p* < 0.001) between sexes in response to the administration of FG-7142, as well as in the first two periods of time corresponding to DBS-IL (*p* < 0.001).

### 3.3. Spectral composition

Figure 3 and Supplementary Table 1 summarize the statistical results of spectral composition. In males, FG-7142 drastically decreased the slow wave relative power in all channels and increased low theta (2.5–5 Hz) relative power in all structures of the amygdalo-hippocampal network. Faster theta oscillations (5–12 Hz) only increased significantly in the HPCd in response to drug administration. Systemic administration of FG-7142 also induced in males evident delta (1.5–2.5 Hz) and beta (16–30 Hz) increases in BLA, HPCv, and HPCi, without changes in HPCd.

In all cases, DBS-IL reversed the effects of FG-7142 administration on these oscillations in a time-dependent manner, returning to basal values throughout stimulation and during the 300 s post-DBS-IL. For a detailed description of the results in males, see Vila-Merkle et al. (2021).

In the female group, FG-7142 administration only decreased the slow wave band in the dorsal regions, HPCd and HPCi. This decrease was accompanied by a slight increase in the relative power of the low theta in the HPCd, but not the remaining structures. Furthermore, the relative power in the delta, high theta, or beta bands did not change in any region.

The comparative analysis between sexes evidenced significant sex differences. Specifically, in males, HPCv and BLA experienced a markedly higher response to FG-7142, mainly in the delta and beta bands. At the same time, the HPCi was the structure with higher differences in the appearance of low theta.

### 3.4. Characterization of theta segments

**FIGURE 4:**
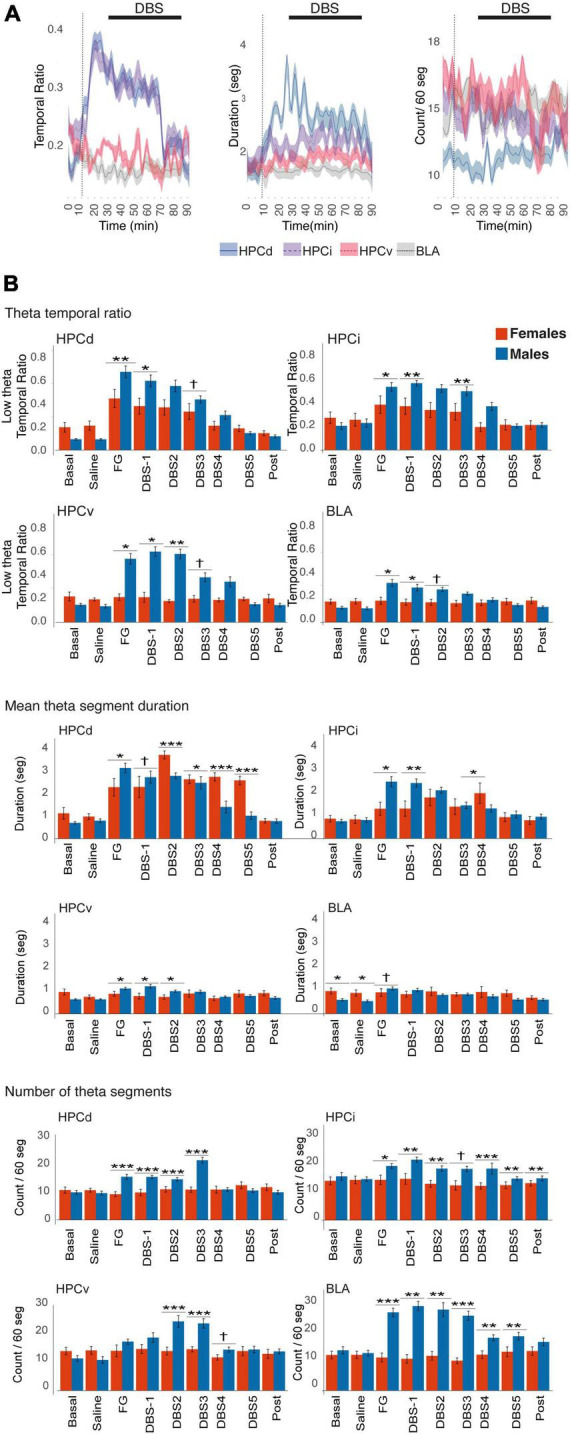
Time-Frequency analysis of the theta activity in females. **(A)** Time course of the theta activity. The black line indicates the DBS-IL. **(B)** Sex differences in theta activity. FG-7142 induced a greater theta band ratio in males, absent in the HPCv and the BLA in females. In females, the segments do not experience noticeable changes in the number of theta segments. The asterisks denote the degree of statistical significance in pairwise comparisons between sexes (***p < 0.001, **p < 0.01, *p < 0.05, and cross: *p* < 0.08). B: basal; S: saline; F: FG-7142; D1-D5: DBS-IL periods 1–5; post: post-DBS; BLA, basolateral amygdala; DBS-IL, deep brain stimulation of the infralimbic cortex; HPCd, dorsal hippocampus; HPCi, intermediate hippocampus; HPCv, ventral hippocampus.

In males, the anxiogenic drug caused a net increase in theta segments in all regions, regarding the number of segments and their duration. In contrast, FG-7142 induced longer theta segments but not its number in females and only in the HPCd, increasing their temporal ratio (Table 2). However, the comparative study between sexes indicates that this temporal ratio occupied by theta segments is lower in females than in males (Figure 4). The more marked differences appeared in the HPCv, where the males reached more than double temporal ratio than females.

**TABLE 2:** Theta segments in females.

	Basal	Saline	FG	DBS1	DBS2	DBS3	DBS4	DBS5	POST
**Theta temporal ratio**									
dHPC	0.194±0.041	0.208±0.039	**0.442**±0.078***	**0.376**±0.069*	**0.365**±0.065**	0.328±0.069	0.208±0.037	0.184±0.027	0.144±0.022
HPCi	0.276±0.049	0.258±0.054	0.388±0.075	0.375±0.070	0.343±0.065	0.327±0.071	0.195±0.039	0.215±0.044	0.214±0.038
HPCv	0.219±0.038	0.194±0.014	0.214±0.028	0.213±0.043	0.180±0.012	0.199±0.028	0.189±0.017	0.199±0.015	0.203±0.015
BLA	0.176±0.020	0.179±0.024	0.183±0.030	0.170±0.027	0.169±0.025	0.162±0.025	0.165±0.025	0.177±0.027	0.184±0.027
**Theta segments mean width**									
dHPC	1.137±0.257	**0.990**±0.128***	**2.303**±0.394***	**2.317**±0.478***	**3.747**±0.178***	**2.765**±0.184***	**2.607**±0.191***	**0.805**±0.101***	0.885±0.101
HPCi	0.885±0.142	0.856±0.282	1.329±0.296	1.337±0.340	**1.834**±0.396***	1.423±0.349	2.023±0.461	0.955±0.200	0.815±0.176
HPCv	0.963±0.133	0.748±0.084	0.884±0.107	0.780±0.128	0.743±0.096	0.896±0.156	0.683±0.097	0.889±0.144	0.911±0.116
BLA	1.005±0.134	0.917±0.136	0.944±0.171	0.866±0.129	0.989±0.193	0.858±0.085	0.965±0.246	0.908±0.136	0.709±0.084
**Number of Segments**									
dHPC	10.720±1.102	10.680±0.718	9.240±0.874	9.840±1.222	11.000±1.017	10.880±0.939	10.880±1.284	12.480±1.211	11.760±1.191
HPCi	14.200±1.409	14.520±1.406	14.560±0.688	14.920±1.929	13.120±1.259	12.560±1.691	12.360±1.152	12.640±1.187	13.360±0.998
HPCv	14.083±1.294	14.333±1.427	14.167±2.088	14.833±1.646	14.083±1.395	14.750±0.938	11.833±0.983	14.083±1.658	13.083±1.630
BLA	12.760±1.356	12.800±1.291	11.840±1.640	11.400±1.555	12.440±1.562	10.680±1.024	12.880±1.367	13.920±1.766	14.160±1.543

Mean ± standard error. Bold: statistical significance; asterisks denote statistical significance between states ***p < 0.001, **p < 0.01, *p < 0.05. BLA, basolateral amygdala; HPCd, dorsal hippocampus; HPCi, intermediate hippocampus; HPCv, ventral hippocampus. FG: FG-7142; POST: post-DBS.

In the BLA, it is noteworthy that females had longer theta segments in the basal state than males. However, none of the manipulations altered any parameter in this region, while in males, the number of theta segments was markedly enhanced.

The application of the DBS-IL in males reversed the altered pattern generated by the anxiogenic drug. In females, this reversion only applied to the HPCd and slightly to the HPCi. In general, there was a reduced response to the DBS-IL in females. However, the duration of theta segments was longer in females than in males, only in the HPCd, in some moments of the stimulation.

### 3.5. Gamma power

**FIGURE 5:**
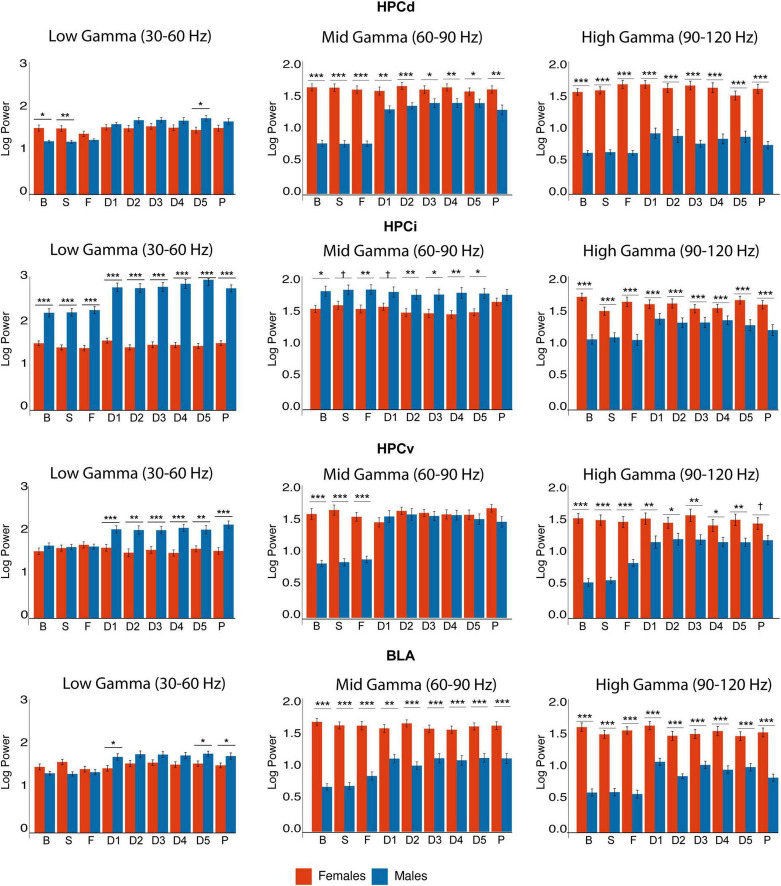
Sex differences in gamma power. Observe the differences in basal gamma activity. All gamma bands are higher in HPCd in females, who also had enhanced high gamma in HPCi and mid and high gamma in HPCv and BLA. In contrast, males had higher mid-gamma in HPCi. Observe the change in gamma activity in males, while females remain stable despite the interventions. Asterisks denote the degree of statistical significance in pairwise comparisons between sexes (***p < 0.001, **p < 0.01, *p < 0.05, and cross: *p* < 0.08). B: basal; S: saline; F: FG-7142; D1-D5: DBS-IL periods 1–5; P: post-DBS. BLA, basolateral amygdala; DBS-IL, deep brain stimulation of the infralimbic cortex; HPCd, dorsal hippocampus; HPCi, intermediate hippocampus; HPCv, ventral hippocampus.

Gamma power (Figure 5 and Supplementary Table 2) exhibited striking differences even in the basal state. Mid- (60–90 Hz) and high- (90–120 Hz) gamma power was higher in females in all regions, while HPCi exhibited remarkable low-gamma (30–60 Hz). In female rats, gamma power did not experience any significant change in any structure, neither in response to FG-7142 nor during the DBS-IL. Minimal intersubject variability and higher power values were observed in all bands. This remarkably greater power in females was significant in all conditions in the comparative study between sexes, even in basal conditions.

In contrast, in males, FG-7142 and DBS-IL exhibited a differential pattern in gamma oscillations in the amygdala and the hippocampal regions. Specifically, FG-7142 enhanced gamma power exclusively in HPCv and amygdala. In the BLA, the carboline augmented the mid-gamma power In the HPCv, FG-7142 enhanced mid- and high-gamma power. However, neither HPCi nor HPCd experienced changes in gamma power in response to the drug. In this case, DBS-IL did not reverse the effects of FG-7142; instead, it even augmented gamma power in the whole circuit, which lasted during the post-DBS period. More specifically, low and high gamma power increased in all structures compared to the baseline and the anxiety-like periods.

### 3.6. Phase locking

**FIGURE 6:**
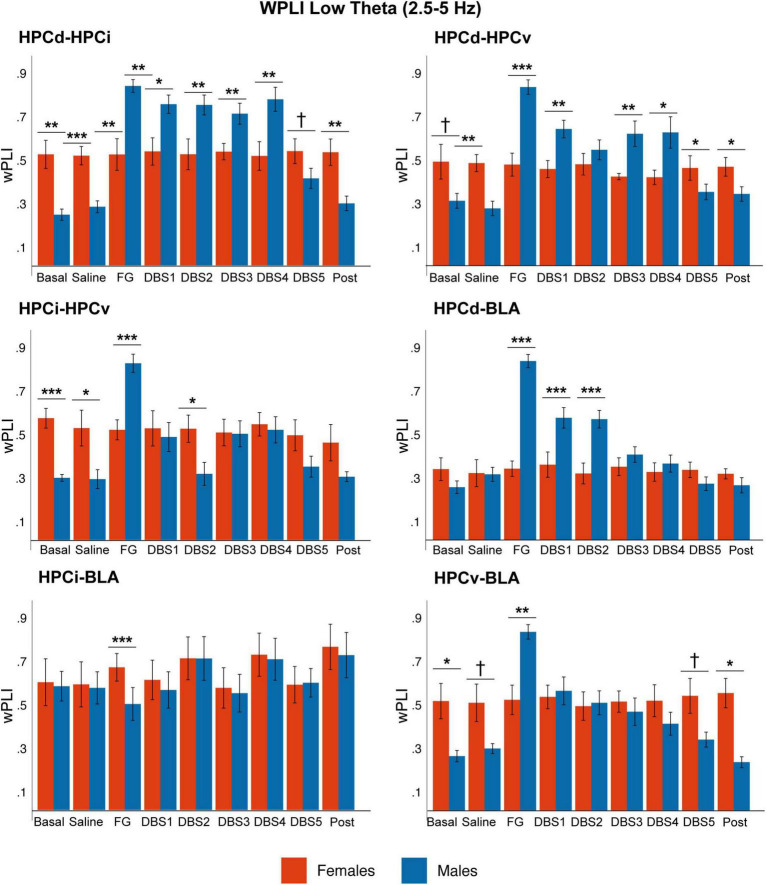
Sex differences in phase-phase coupling (wPLI) in the low theta band. While in males, the phase-coupling increased significantly in response to FG-7142 among all circuit structures and decreased with DBS-IL, it remained constant in time in females. Note the higher coupling between HPCd-HPCi, HPCd-HPCv, and HPCi-HPCv in the basal anesthesia condition in females. The asterisks denote the degree of statistical significance in pairwise comparisons by sex (***p < 0.001, **p < 0.01, *p < 0.05, and cross: *p* < 0.08). B: basal; S: saline; F: FG-7142; D1-D5: DBS-IL periods 1–5; P: post-DBS. BLA, basolateral amygdala; DBS-IL, deep brain stimulation of the infralimbic cortex; HPCd, hippocampus dorsal; HPCi, intermediate hippocampus; HPCv, ventral hippocampus.

We analyzed the degree of phase-locking between regions in the slow wave, delta, low theta, and beta bands by measuring the WPLI. Figure 6 and Supplementary Table 3 summarize the statistical values.

In basal conditions, males exhibited high coupling in the slow wave band in all regions. However, the anxiogenic drug increased WPLI at low theta frequencies among all hippocampal subregions (HPCd-HPCi, HPCi-HPCv, and HPCd-HPCv), between HPCd-BLA and HPCv-BLA. In addition, the most ventral regions of the network (HPCv-BLA, HPCi-BLA, and HPCv-HPCi) increased the phase-locking at delta and beta frequencies. The DBS-IL returned all these values to the basal conditions.

The results obtained in this analysis showed that during the anxiety-like state, the synchronization increased in different subnetworks within the amygdalo-hippocampal circuit at delta, theta, and beta frequencies, which DBS-IL was capable of reversing.

In contrast, females did not reach statistical differences in any pair of the network. The comparative analysis between sexes highlighted that, in basal anesthesia conditions, there was a significant different coupling between some structures. Females showed a lower basal coupling at slow waves and a higher coupling at low theta values. In contrast, males have a stronger response to FG-7142.

### 3.7. Mutual information

**FIGURE 7:**
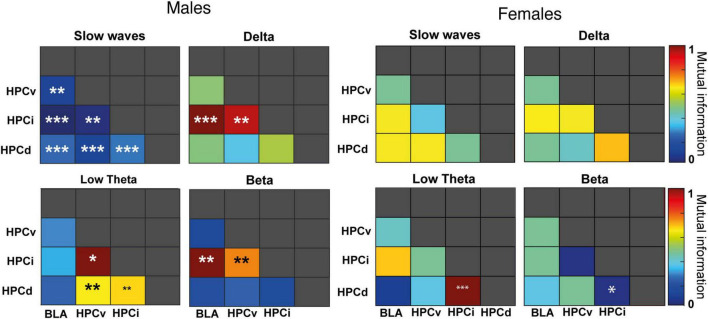
Sex differences in mutual information. Pairwise diagram showing the change in mutual information (normalized to 0–1) following FG-7142 administration. The asterisks denote the degree of statistical significance compared to the basal state (***p < 0.001, **p < 0.01, *p < 0.05).

Our results in MI (Figure 7 and Supplementary Table 4) also highlighted a more robust effect of FG-7142 in network communication in male than in female rats. In males, FG-7142 significantly reduced the slow-wave MI among all structures (*p* < 0.01). The MI also indicated a differentiated pattern in the dorsoventral axis. Specifically, the FG-7142 significantly increased the interaction between all hippocampal areas (HPCd-HPCi, HPCd-HPCv, and HPCi-HPCv) in the low theta band. Instead, during this state, it also caused an increase in the communication between HPCi-BLA and HPCi-HPCv at delta and beta frequency bands. The MI in the different frequency bands generated by the FG-7142 returned to basal values as DBS-IL progressed.

In contrast, in females, only the communication between HPCd-HPCi had significant differences following the administration of FG-7142. Between these regions, MI increased at low theta (*p* < 0.001) and decreased at beta (*p* < 0.05) frequency. The communication exchange between the other structures of the network did not change statistically. Moreover, despite the slight effects observed, the DBS-IL did not cause any significant impact relative to baseline periods between any of the structures registered to any frequency band.

Again, the comparative analysis between both sexes (Supplementary Table 4) evidenced a sexually differentiated communicative pattern in the amygdala-hippocampal circuit, not only to the drug but also in basal conditions. During the basal period, females had lower mutual information between HPCi-BLA at slow waves and higher at low theta between HPCd-HPCi, HPCv-HPCi, HPCi-HPCv, and HPCv-BLA. Also, the FG-7142 provoked a differentiated response in the communication in slow waves in most pairs, given the lack of response in females. Concerning the response to the DBS-IL, the most notable differences were observed in the interaction between HPCi-BLA in slow waves communication, with males always showing higher values. In contrast, females had higher values in theta communication between HPCd-HPCi, HPCd-HPCv, and HPCv-BLA.

### 3.8. Phase-amplitude coupling

**FIGURE 8:**
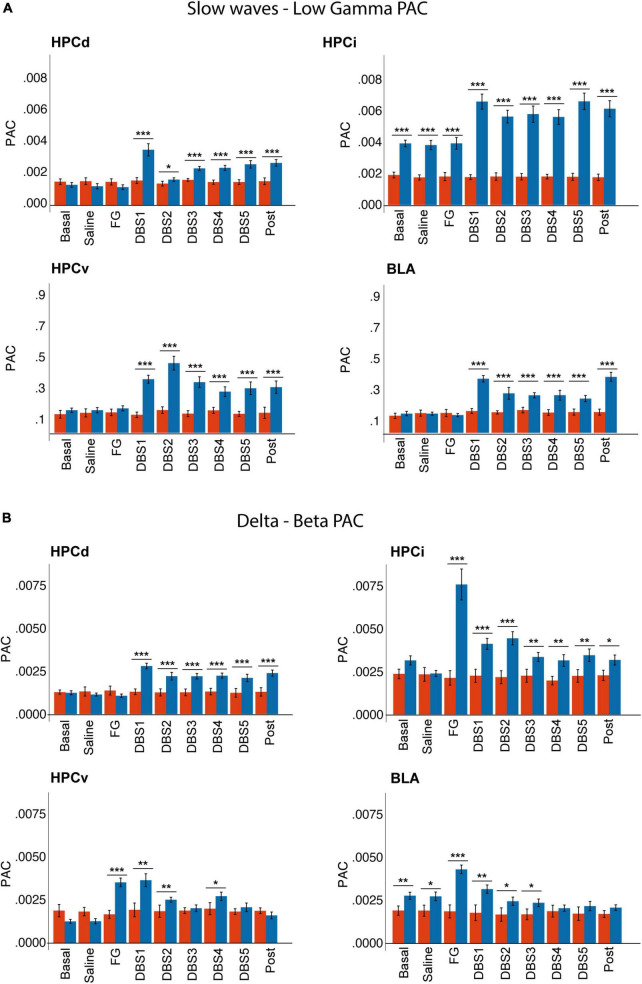
Sex differences in phase-amplitude coupling between **(A)** slow waves and gamma and **(B)** delta and beta bands. Note that males showed a higher delta-beta coupling in response to FG-7142, restored by the DBS. Also, only males had increased slow waves-gamma coupling in response to the DBS. Note that males already had higher basal slow waves-gamma coupling in HPCi and delta-beta coupling in BLA. ***p < 0.001; **p < 0.01; *p < 0.05.

Our results highlight a different degree of synchronization between the slow wave-theta, delta-beta, and theta-gamma frequency bands in both sexes (Supplementary Table 5 and Figure 8).

In males, the anxiogenic drug increased delta-beta PAC in HPCi, HPCv, and BLA, while theta-gamma coupling increased only in the HPCi. DBS-IL reversed the changes in delta-beta coupling. Instead, its application caused a significant increase between slow waves and the gamma band, as well as in theta-gamma coupling.

In females, the cross-frequency coupling did not show significant variation in response to FG-7142 or DBS-IL. The comparison between sexes reflected the different reactions to both experimental conditions, with effects in males but not females. Females had lower slow waves-gamma and theta-gamma coupling in HPCi than males in all states, and females also had a lower delta-beta coupling in BLA since the basal period. Also, only males had enhanced slow waves-gamma and theta-gamma in all regions during the DBS-IL application.

### 3.9. Neuronal activation by c-Fos expression

The analysis of neuronal activation on a broader network complemented the electrophysiological study of the amygdalo-hippocampal circuit. To do this, we quantified c-Fos protein expression, in a total of 47 encephalic areas, in male and female rats, under different experimental conditions: (1) only with the surgical implantation procedure of the stimulation electrode (sham group); (2) with one h of DBS-IL (DBS group); (3) with electrode implantation and FG-7142 administration (experimental group sham-FG); and (4) with the simultaneous combination of 1-h DBS-IL and FG-7142 administration (FG-DBS group). Five animals were used for each experimental condition and sex. The measurement was performed in the neocortex, septal region, hippocampal formation, amygdalar complex, and diencephalic and brainstem levels.

**FIGURE 9:**
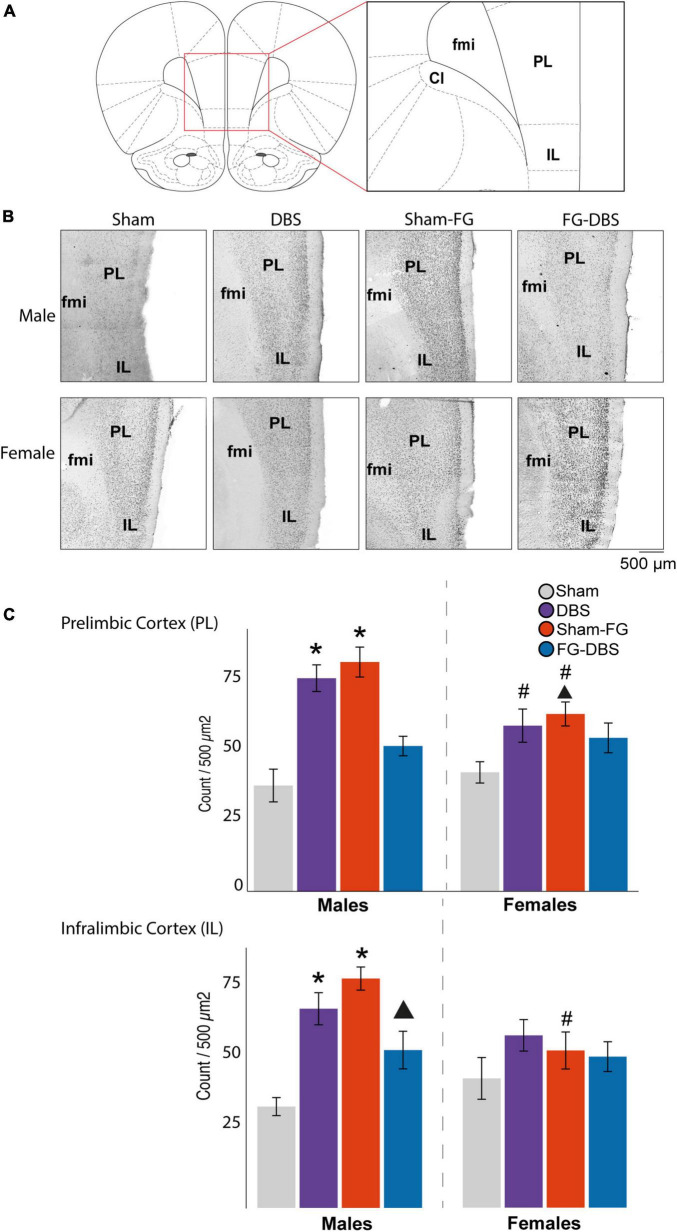
Sex differences in c-Fos labeling in IL and PL in response to FG-7142 and DBS-IL. **(A)** Diagram of the regions used for the cell count. **(B)** Representative photomicrograph. **(C)** Statistical results. In males, the administration of FG-7142 (sham-FG group) and, to a lesser extent, DBS-IL (DBS group) increased the immunoreactivity in IL and PL with significant differences compared to the sham and FG-DBS group. Drug administration followed by DBS-IL (FG-DBS group) in males showed a reduced immunoreactivity, although with significant differences in PL compared to the sham group. Both the application of DBS-IL and FG-7142 administration elevated c-fos expression in IL and PL in females. However, this increase showed only a statistical trend in IL compared to the sham group. The comparative analysis revealed the existence of sex differences in sham-FG (*p* < 0.05) in both IL and PL. Asterisk denotes pairwise comparison compared to the sham group in the Kruskal–Wallis test between experimental groups (*p* < 0.05). Triangle denotes *p* < 0.08 in the same test. ^#^Denotes statistical significance (*p* < 0.05) in the Mann–Whitney test between sexes. fmi, forceps minor of the corpus callosum; IL, infralimbic cortex; PL, prelimbic cortex.

**FIGURE 10:**
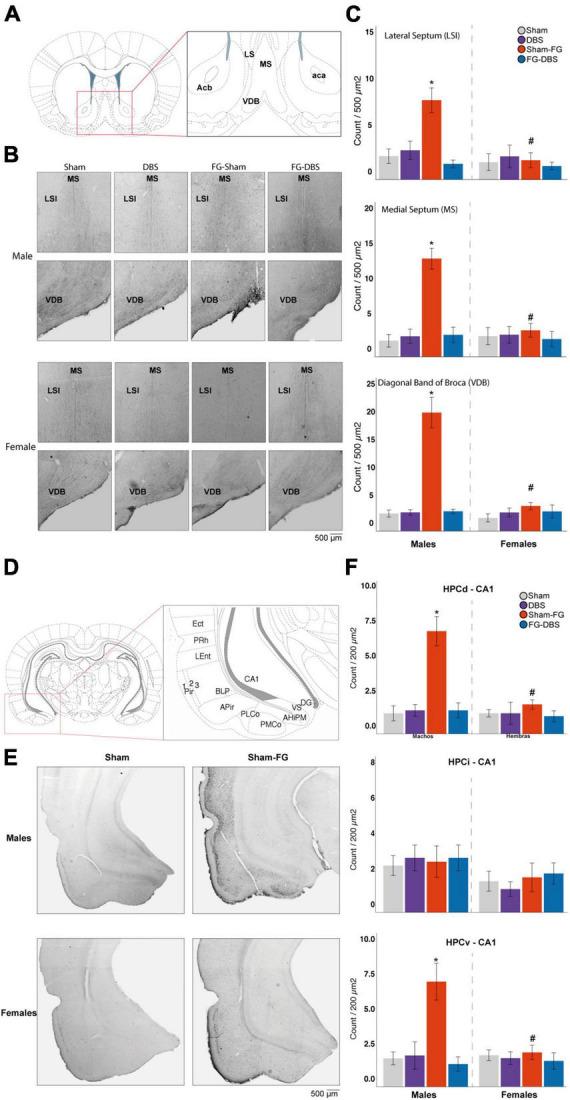
Sex differences in c-Fos labeling in the septal area and the hippocampus. **(A)** Schematic diagram of septal regions used for cell count. **(B)** Representative photomicrographs in male and female specimens. **(C)** Statistical values in both sexes. In males, FG-7142 administration increased immunoreactivity in the LSI, the MS, and the VDB, with significant differences compared to the other experimental conditions. The combination of the anxiogenic drug and the DBS-IL (FG-DBS group) in males showed a similar immunoreactivity to the sham group. In females, no significant differences were found. The comparative analysis revealed the existence of sex differences in the sham-FG group (*p* < 0.05) in these three structures. **(D)** Schematic diagram of hippocampal regions used for cell count. **(E)** Representative photomicrographs in the HPCv of male and female specimens. **(F)** Statistical values in both sexes. In males, FG-7142 administration increased immunoreactivity in the CA1 of HPCd and HPCv, with significant differences compared to the other experimental conditions. The combination of the anxiogenic drug and the DBS-IL (FG-DBS group) in males showed a similar immunoreactivity to the sham group. In females, no significant differences were found. The comparative analysis revealed the existence of sex differences in the sham-FG group (*p* < 0.05) in these three structures. *Statistical significance (*p* < 0.05) in the Kruskal–Wallis test comparing experimental groups. ^#^Denotes statistical significance (*p* < 0.05) in the Mann–Whitney test between sexes. CA1, Cornu Ammonis field 1; Ect, ectorhinal cortex; HPCd, dorsal hippocampus; HPCi, intermediate hippocampus; HPCv, ventral hippocampus; LSI, intermediate lateral septum; MS, medial septum; VDB, diagonal band of Broca, vertical limb; Pir, piriform cortex; PMCo, posteromedial cortical amygdaloid nucleus; PLCo, posterolateral cortical amygdaloid nucleus; PRh, perirhinal cortex; LEnt, lateral entorhinal cortex.

**FIGURE 11:**
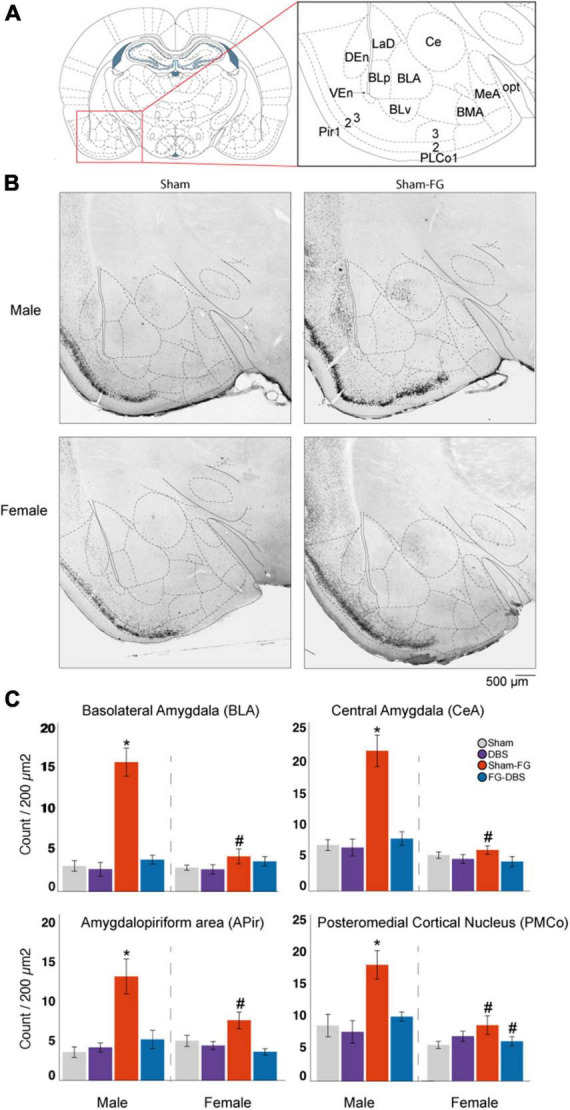
Sex differences in c-Fos labeling in the amygdalar complex. **(A)** Schematic diagram of the regions used for cell count. **(B)** Representative photomicrographs in male and female specimens. **(C)** Statistical values in both sexes. In males, FG-7142 increased significantly c-Fos expression in the BLA, CeA, Apir and PMCo. Administration of FG-7142 followed by DBS-IL (FG-DBS group) in these regions showed similar immunoreactivity to the sham and DBS group. In females, although FG-7142 produced a slight increase in c-Fos levels, these were not significant. The comparison between sexes revealed differences in the sham-FG group in BLA, CeA, Apir and PMCo. *Denotes statistical significance (*p* < 0.05) in the Kruskal–Wallis test between the sham, DBS, sham-FG and FG-DBS groups. ^#^Denotes statistical significance (*p* < 0.05) in the Mann–Whitney test between sexes. BLA, basolateral amygdala; BMA, basomedial amygdala; CeA, central amygdala; LA, lateral amygdala; NE, endopiriform nucleus; Pir, piriform cortex; PLCo, posterolateral cortical amygdaloid nucleus; PMCo, posteromedial cortical amygdaloid nucleus.

**FIGURE 12:**
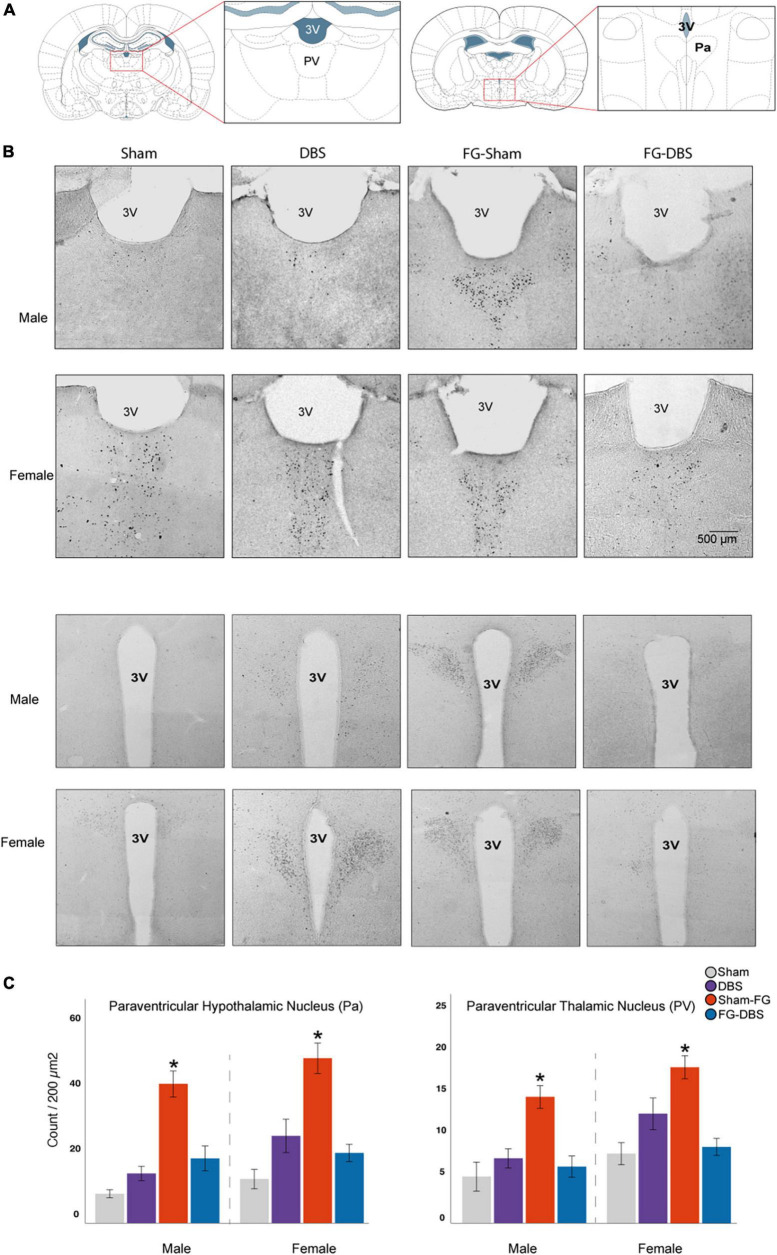
Sex differences in c-Fos labeling in the paraventricular thalamic and hypothalamic nuclei. **(A)** Schematic diagram of the regions used for cell count. **(B)** Representative photomicrographs in male and female specimens. **(C)** Statistical values in both sexes. Although females had higher values in basal and DBS conditions than males, the differences were not significant. In both sexes, FG-7142 increased c-Fos expression in the PaaV and PV nuclei, and the combination of the drug and the DBS (FG-DBS group) returned to similar levels than the sham group. *Denotes statistical significance (*p* < 0.05) in the Kruskal–Wallis test between the sham, DBS, sham-FG and FG-DBS groups. 4V: fourth ventricle; Pa: paraventricular hypothalamic nucleus; PV: paraventricular thalamic nucleus.

**FIGURE 13:**
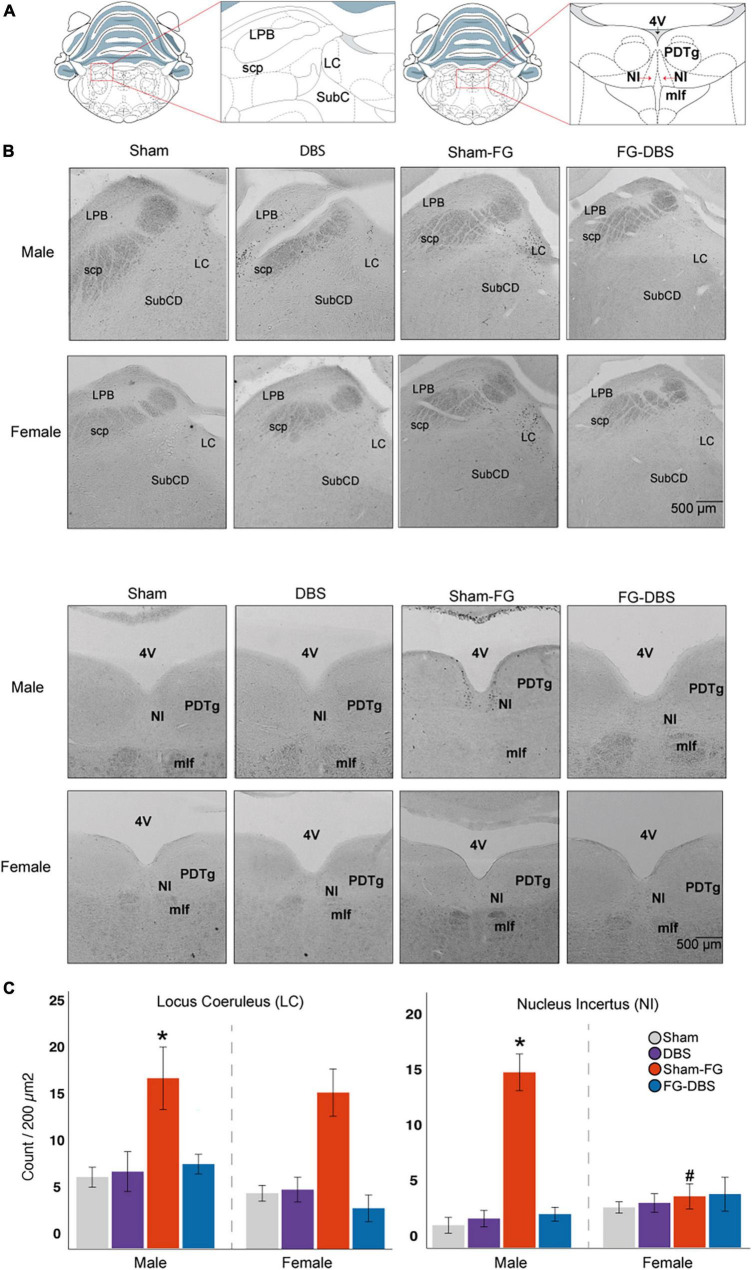
Sex differences in c-Fos labeling in brainstem nuclei. **(A)** Schematic diagram of the regions used for cell count. **(B)** Representative photomicrographs in male and female specimens. **(C)** Statistical values in the DR, LC, and NI in both sexes. While FG-7142 increased c-Fos expression in the LC in both sexes, cell count only raised in the NI in males. The comparison between sexes evidences a distinct response to the drug. The combination of FG-7142 and DBS-IL restored basal values. *Denotes statistical significance (*p* < 0.05) in the Kruskal–Wallis between the sham, DBS, sham-FG, and FG-DBS groups. ^#^Denotes statistical significance (*p* < 0.05) in the Mann–Whitney test between sexes. DR, dorsal raphe; LC, locus coeruleus; NI, nucleus incertus.

Supplementary Table 6 include all the statistical comparisons six derived from the Kruskal–Wallis test for comparing experimental groups within the same sex and the statistical test of Mann–Whitney for evaluating sexual differences in every experimental condition. Figures 9–13 illustrate the results in each region.

Our results show a greater c-Fos expression at telencephalic levels, including cortical areas, the septal region, the hippocampal formation and the amygdalar complex. A similar response, however, was observed in diencephalic and brainstem nuclei related to stress, except for the nucleus incertus.

#### 3.9.1. Cortical structures

Figure 9 shows the microphotographs of a representative case and statistics of IL and PL in males and females.

In IL, DBS only caused a significant increase (*p* < 0.05) of c-Fos expression in males. In addition, in these animals, the application of DBS-IL (DBS group) significantly increased immunoreactivity, practically doubling in prelimbic (PL), cingulate, and granular insular cortices compared with the sham group. In contrast, the DBS-IL did not induce significant differences in any analyzed cortical structures in females.

In males, the administration of the drug FG-7142 (sham-FG group) also caused a significant increase in cell reactivity in most cortical structures, with higher expression levels than with the DBS. Specifically, the expression was highest in ectorhinal, entorhinal, IL, PL, insular cortex, and ventral tenia tecta, followed by cingulate, piriform, and peduncular cortices compared to the sham animals. The male group receiving the combination of the anxiogenic drug and the DBS-IL (FG-DBS group) reached similar values of c-Fos expression to the sham group in all structures. However, no significant variations were observed in any experimental condition in the orbital cortices or the dorsal tenia tecta.

These results were different from those observed in the female group. In this case, although both the DBS group and the sham-FG group slightly increased the c-Fos expression in most cortical structures, these were not statistically different and only presented a trend in PD and IL. In IL, the highest c-Fos protein expression corresponded to the administration of FG-7142 (*p* < 0.08 compared to the sham group).

The comparison between males and females for each condition was evaluated through the Mann–Whitney statistical test, which revealed the existence of sexual differences (*p* < 0.05). Among the sham groups, the ventral tenia tecta already showed a higher reactivity in females. This region showed differences in all experimental conditions except in response to FG-7142. Cg and IL also showed differences in response to DBS-IL alone, with a significantly higher expression in males.

Main differences in the labeling arose in the sham groups receiving FG-7142, with the cingulate, ectorhinal, entorhinal, insular, IL, and PL presenting a significantly lower expression in females. In contrast, animals that received the FG-DBS combination did not show significant differences, with values always similar to those of the sham group. The only exception was the ventral tenia tecta, which was already different in the sham groups.

#### 3.9.2. Septal area

Figure 10 subplots (A)–(C) summarize the results in the septal area. The immunoreactive cell count was carried out in the lateral septum at the dorsal (LSD), intermediate (LSI), and ventral level (LSV), the medial septum (MS) and in Broca’s diagonal band (VDB).

In males, neither the electrode implantation procedure nor the DBS-IL caused statistically significant changes in any regions of the septal area. Likewise, animals that received the FG-DBS combination presented values similar to the sham group. No changes were observed in activation levels in response to FG-7142 administration in LSD or LSV. However, FG-7142 alone induced cellular activation in the LSI, the MS, and the VDB, annulled by the combined administration of the drug with the DBS-IL.

In contrast, in females, slight c-Fos increases were observed in the LSI following the DBS-IL and the MS and in the VDB in response to FG-7142. However, the differences were not statistically significant compared with the other experimental conditions.

The Mann–Whitney test corroborated this differential response, revealing a higher reaction to the anxiogenic drug in the LSI, MS, and VDB regions in males (*p* < 0.05) but not in the remaining experimental conditions.

#### 3.9.3. Hippocampal formation

Figure 10 subplots (D)–(F) summarize the results in the hippocampal formation. The selected areas in the hippocampal formation corresponded with dorsal and ventral DG, CA1, CA2, and CA3 fields and the intermediate CA1 field and DG.

In females, there was no significant difference in any region. In males, the anxiogenic drug increased c-Fos expression (*p* < 0.05) in the CA1 cells of the HPCd and HPCv, reversed by the combination of FG-7142 and DBS-IL. However, in males, no significant differences arose in any condition in the DG, CA2, and CA3 fields of HPCd or HPCv, nor the HPCi. Furthermore, DG immunoreactivity in the HPCi was null in all experimental conditions in both sexes.

The comparative study between sexes corroborated the existence of sex differences in the different manipulations. Females receiving DBS alone had lower activation in the HPCv-CA2. In response to FG-7142, males also showed a higher expression in dorsal and ventral CA1 fields and dorsal DG (*p* < 0.05). Finally, females also showed lower expression in the group receiving the combination of FG-7142 and DBS in the dorsal CA3 and DG and ventral CA2.

#### 3.9.4. Amygdalar complex

Figure 11 shows representative photomicrographs of the amygdalar complex and the statistical results. We measured c-fos immunoreactivity in BLA, BMA, CeA, MeA, LA, the amygdalopiriform transition zone (Apir), the cortical nucleus of the amygdala at posterolateral (PLCo) and posteromedial (PMCo) levels, the amygdalohippocampal region (AHi) and in the endopiriform nucleus (NE).

In the female group, BLA, CeA, Apir, PMCo, and NE showed a slight increase in the c-Fos expression in the sham-FG animals but without statistical significance. In males, the application of DBS-IL alone did not cause any statistically significant change compared to the sham group in none of the regions of the amygdala complex. However, the administration of the anxiogenic drug induced the elevation of c-Fos protein expression (*p* < 0.05) in BLA, CeA, MeA, Apir, and PMCo. The combination of FG-7142 and DBS-IL reversed this activation to basal levels.

The comparative study between both sexes confirmed the differences in all the experimental conditions, even between the sham groups. Females showed a lower expression than males in BMA, MeA, and AHi (*p* < 0.05). Similarly, the response to DBS-IL showed significant differences between both sexes in MeA and PLCo, again with a lower expression in females. Regarding the sexual differences in the expression of c-Fos after the anxiogenic drug administration, significant differences appeared in almost all the regions: BLA, BMA, CeA, MeA, Apir, PLCo, and PMCo had lower levels of reactivity in female rats. Finally, in the FG-DBS groups, sex differences arose in CeA, MeA, PLCo, PMCo, and AHi, again with lower immunoreactivity levels in females.

#### 3.9.5. Diencephalon

Measurement of the c-Fos protein was carried out in the centromedian nucleus (CM), paraventricular (PV), reuniens (Re), and rhomboid (Rh) thalamic nuclei, and the paraventricular hypothalamic nucleus (Pa). Figure 12 illustrates the results in paraventricular nuclei.

Unlike at telencephalic levels, we observed a similar activation in both sexes. In males and females, the administration of FG-7142 induced very high levels of expression of c-FOS compared to the other experimental conditions in the PV and the Pa but not in the remaining diencephalic structures, in which no significant results were obtained. In addition, in both sexes, the administration of the anxiogenic drug followed by the application of DBS-IL reversed this effect, with similar levels to the sham or DBS group.

Accordingly, the comparative study confirmed this similar behavior in both sexes. None of the diencephalic regions showed significant sex differences in response to none of the evaluated manipulations.

#### 3.9.6. Brainstem

In the brainstem, the cell count was carried out in the dorsal raphe (DR), the locus coeruleus (LC), the nucleus incertus (NI), the lateral parabrachial nucleus (LPB), and the periaqueductal gray (PAG). Figure 13 shows the statistical results and photomicrographs at these levels.

The DBS-IL alone did not cause any activation over the sham group. In contrast, the animals receiving FG-7142 of both sexes showed a significant increase in the labeling in the DR and LC compared to the other conditions. However, only males increased c-Fos expression in NI with the anxiogenic drug. In addition, males showed significantly enhanced immunoreactivity in the DR and the NI.

In all cases, the combined application of the anxiogenic drug and DBS-IL showed similar levels of cell labeling to the sham group and the DBS group. In females, animals receiving both FG-7142 and the DBS had significantly minor cell activation in LC.

## 4. Discussion

In this work, we compared the effects of FG-7142 administration in urethane-anesthetized male and female rats. The study complements prior observations only in male rats (Vila-Merkle et al., 2021). This drug has proven to have anxiogenic effects in humans and experimental models in various pharmacological, metabolic, neurochemical, and behavioral tests (Evans and Lowry, 2007). We found remarkable differences between both sexes, having much more intense effects in males.

In male rats, FG-7142 administration suppressed the characteristic slow wave pattern of the anesthesia and increased the peak frequency in all structures to the range of low theta or type 2 theta. This frequency increase was less marked in females. In this case, the peak frequency was around 1.2 Hz, in the delta range, and only significant in the dorsal and intermediate regions of the hippocampus.

Furthermore, only in males FG-7142 induced the appearance of information flows with high synchronization at low theta frequencies between the hippocampal regions. At the same time, the ventral amygdalohippocampal subcircuits exchanged information predominantly at coupled delta and beta frequencies. Females had a distinct communication pattern in the basal state which could prevent the alterations induced by the drug.

Finally, in both sexes, the drug increased c-Fos expression in brainstem structures related to arousal and stress like LC and DR, but NI activation only happened in males. In both sexes, at diencephalic levels, FG-714 hyperactivated the hypothalamic and thalamic paraventricular nuclei, highly related to behavioral control. Nevertheless, activation of the septal area, amygdala, hippocampus, and neocortical levels only occurred in the male group.

Our results in males, correlate well with the existing literature on the effects of the drug. FG-7142, in males, at similar and even lower doses than that used in this work induces pronounced anxiogenic effects on standardized tests such as the open field (Stephens et al., 1987; Dawson et al., 2006), the elevated plus maze (Pellow and File, 1986; Rodgers et al., 1995; Atack et al., 2005; Dawson et al., 2006), or the darkness-light test (Bueno et al., 2005). FG-7142 also alters the ability to regulate and inhibit emotional responses, potentiates fear behaviors, and induces avoidance deficits (Thiébot et al., 1991; Harris and Westbrook, 1998; Jelen et al., 2003; Kim and Richardson, 2007; Willadsen et al., 2018) and learned helplessness (Weiss et al., 1981; Drugan et al., 1985; Short and Maier, 1993; Maier and Watkins, 2005).

FG-7142 increases corticosterone levels in plasma (Pellow and File, 1985; Stephens et al., 1987) and activates brain structures involved in stress, including the amygdala, the HPCv, the bed nucleus of the stria terminalis, and the cingulate, prelimbic and IL cortices, and brainstem nuclei including the LC, the PAG, NI, or DR (Corda et al., 1983; Lyss et al., 1999; Singewald and Sharp, 2000; Singewald et al., 2003; Abrams et al., 2005; Funk et al., 2008). In turn, Hackler et al. (2007) observed an increase in the BOLD signal in the amygdala, the HPCd, and the hypothalamus in male rats for at least 40 min after administration of the drug. This latter effect matches the results of previous work in male rats that received FG-7142, in which the duration of the effect was more than 90 min without any spontaneous reversal observed (Vila-Merkle et al., 2021).

At the behavioral level, evidence supports the existence of sex differences in rodents in response to FG-7142. Early experiments (Meng and Drugan, 1993) showed that while male rats significantly decreased male open-field exploratory activity with FG-7142 at 5, 10, and 20 mg/kg, female rats needed a higher dose for lowering open-field activity (40 mg/kg). In contrast, Cottone et al. (2007) found that female rats with FG-7142 at the dose used in the present study (7.5 mg/kg) had anxiogenic-like behavior in the elevated plus-maze. Sex differences have also been described in other anxiety and depression models (Kokras and Dalla, 2014; Pavlova et al., 2020). In studies comparing female vs. male rats in the elevated plus maze, female rodents exhibit less anxiety-like behavior (Mora et al., 1996; Díaz-Véliz et al., 1997; Fernandes et al., 1999; Walf and Frye, 2007).

A major concern can be the possible influence of the hormonal stage in anxiety-like behavior. Previous findings suggest that proestrus or estrus may be anxiolytic (Mora et al., 1996; Díaz-Véliz et al., 1997; Fernandes et al., 1999; Walf and Frye, 2007). However, these concerns do not correspond to more recent observations made by Scholl et al. (2019). They aimed to clarify conflicting data regarding the anxiety-like behavior of female rats and the influence of the estrous cycle. Their study compared the behavior of male and estrous-staged female rats the elevated plus maze, open field, and a social interaction/avoidance paradigm. Regardless of the estrous cycle, female rats in the elevated plus maze spent more time in open arms, indicating lower anxiety. In the open field and the social anxiety test, however, they found no differences between males and females, and again, they found no influence of the estrous cycle.

In our experiments, the lack of control of the estrous phase could a limitation of our study to be considered in further studies. A detailed analysis of the effects of FG-7142 according to the estrous phase could clarify anxiety processing in female rats. However, not only female hormones influence anxiety-like behavior. Testosterone also contributes to sex differences in anxiety-like measures. Younger to middle-aged male rats, with higher testosterone levels, exhibit more anxiety-like behavior than females (Domonkos et al., 2018).

One of the most evident findings in this work is that during the anxiety-like states, slow-wave activity significantly decreased in all regions in males and only in the HPCd and HPCi in females. In both cases, acute application of DBS-IL restored the oscillatory pattern in every altered structure. Previous studies by our group have already demonstrated the effect of DBS-IL on slow waves. DBS-IL increased its power in HPCd and the BLA in anesthetized and in HPCv in FG-7142-treated male rats (Cervera-Ferri et al., 2016; Vila-Merkle et al., 2021). Etievant reported a similar effect in the infralimbic cortex of non-anesthetized rats after the DBS (Etiévant et al., 2015). In the present study, however, FG-7142 did not suppress the slow waves in the amygdala nor the HPCv in females. This different response can be related to a different cognitive response to stress paradigms. A distinct behavioral and cognitive response to stress has been observed in female rats, even with enhanced performance (McLaughlin et al., 2007; Bowman and Kelly, 2012).

Slow waves involve the synchronization of the membrane potentials of large distributed neuronal populations located mainly in the cerebral cortex and subcortical structures. These oscillations induce alternation between largely hyperpolarized states, characterized by neuronal silencing and depolarization periods (Van Someren et al., 2011). In mammals, including humans, the origin of slow waves seems to be cortical, since its generation depends on regions located in the PFC and anterior cingulate cortex, from where they are distributed to the rest of the brain in the anteroposterior direction (Massimini et al., 2004; Murphy et al., 2009). Slow waves are relevant to the maintenance of the general synchronization of the network, biochemical homeostasis of neurons, adaptation to environmental pressure, and memory consolidation (Maquet et al., 1997; Esser et al., 2007; Riedner et al., 2007; Neske, 2016).

The change from low-frequency oscillations to a sustained theta state suggests a general activation of the circuit in males and only in the dorsal and intermediate hippocampus in females. Theta activity is known for its involvement in the active states of the hippocampal formation, allowing memory processing (Hasselmo, 2005; Colgin, 2013) with dissociable oscillatory components for spatial and emotional cues (Wells et al., 2013). In rodents, theta has been subdivided into two bands: type 1 or non-cholinergic theta (referred to as high theta in this paper) and type 2 or cholinergic theta (referred to as low theta in this work).

Our results in males are consistent with previous studies finding theta type 1 rhythm mainly in the HPCd (Buzsáki and Moser, 2013), while type 2 theta activity depends mainly on HPCv (Gray, 1982; Kjelstrup et al., 2002; Pentkowski et al., 2006; Dringenberg et al., 2008). High theta or type 1 theta is related to sensorimotor integration (Bland and Oddie, 2001), locomotion (Vanderwolf, 1988; Fuhrmann et al., 2015), navigation, and spatial learning (Winson, 1978), while low or type 2 theta plays an important role in arousal, immobility and emotional behaviors, including defense, stress (Hegde et al., 2008; Jacinto et al., 2013, 2016), fear (Narayanan et al., 2007; Lesting et al., 2011; Seidenbecher and Lesting, 2012), and anxiety (Adhikari et al., 2010; Jacinto et al., 2013).

There is evidence supporting that theta activity in the HPCv is necessary for behavioral control during situations requiring high levels of arousal and anxiety in rodents, such as exploring unsafe places, the presence of predators, or decision-making in conflict situations (Bannerman et al., 2004; Adhikari et al., 2010; Seidenbecher and Lesting, 2012). Male rats familiar with an environment show decreased low theta rhythm in HPCd, HPCv, and BLA. However, anxious rats exposed to a novel environment exhibited increased theta power significantly in HPCv and BLA, and although to a lesser extent also in the HPCd (Jacinto et al., 2013), indicating that the increase in theta rhythm in these areas was related to the increase of anxiety levels. In contrast, reduced hippocampal theta activity is related to low-anxiety states and behavioral disinhibition while improving active avoidance behaviors in conflict-generating tasks (McNaughton et al., 2013). In addition, anxiolytic drugs reduce the frequency, power, and amplitude of the hippocampal theta rhythm, either under conditions of anesthesia or free movement (Zhu and McNaughton, 1994; McNaughton et al., 2007; Yeung et al., 2012).

FG-7142 induced an attentional or hyperarousal-like state, in which the activated amygdalo-hippocampal circuit appears to be processing the anxiogenic stimulus, with a very stable and sustained low-frequency theta. Although theta activity is necessary for the proper functioning of the hippocampus in memory and attention management, the dynamic changes of the oscillation are necessary for processing incoming information (Pomper and Ansorge, 2021). So, an excessively sustained theta could imply a block for processing other incoming stimuli. The cessation of theta activity is necessary to allow a change in focus of attention to switch attention (Kitchigina et al., 1999). A similar state arises following corticotropin-releasing hormone infusion into the dorsal raphe nucleus, which can be identified as a stress signal (Merino et al., 2021). This sustained activity could avoid the correct processing of new information and contribute to cognitive deficits associated with anxiety and comorbid major depression.

However, the different dorsoventral responses in females should be addressed in further studies to better delineate the behavioral correlate in stress management. The anxiogenic drug only increased theta activity in the HPCd. These results coincide with recent studies (Horváth et al., 2015; Ou et al., 2019) carried out in rats and mice of both sexes, observing a sexually differentiated response in the elevated plus maze. The authors found an increase in theta activity of HPCd in males and females, despite females showing lower anxiety-related symptoms in general. Their results would indicate that theta activity in the HPCd in females would not reflect a sustained state of anxiety without the presence of such activity in HPCv and BLA. This hypothesis is supported by the fact that optogenetic activation and inhibition of the BLA-HPCv projections increase and decrease anxiety-related behaviors in rodents (Felix-Ortiz et al., 2013).

In both sexes, the DBS-IL disrupted the persistent theta oscillations gradually causing their disappearance and returned the network to the slow-wave profile. It should be noted that depressed patients and animal models of depression exhibit a decrease in slow waves (Benca et al., 1992; Armitage, 1995; Dijk, 2010; Duncan and Zarate, 2013). Also, antidepressant therapies such as transcranial magnetic stimulation (Marshall and Binder, 2013; Binder et al., 2014) and antidepressant drugs not only increase the slow oscillations in the amygdalo-hippocampal-prefrontal circuit but also improve the management of cognitive and emotional information (Argyropoulos et al., 2009; Landsness et al., 2011; Duncan et al., 2013). Therefore, the action of the DBS-IL would suppress the pathological activity, reversing its effect by reinstating slow wave oscillatory activity, leaving the hippocampus and amygdala available for cognitive and emotional processing. However, since most works have been carried out in male rats, our work raises questions about the different oscillatory activity between both sexes.

Another relevant finding of this work was the sexually differentiated gamma activity in all experimental conditions. Females had much higher levels than males of basal gamma activity in all regions, coupled with slow waves and theta, and neither the administration of FG-7142 nor the DBS-IL induced a significant increase in gamma power in this group. In contrast, in male rats, with a lower basal gamma, the drug enhanced this activity in the more ventral regions, BLA and HPCv, and the DBS induced an even higher increase of gamma in all structures.

Gamma waves are fast-frequency oscillations which reflect the neuronal activity at the local level (Ray et al., 2008; Sirota et al., 2008). Clinical studies carried out with depressed and anxious patients (Lee et al., 2010; Fitzgerald and Watson, 2018) and animal models of depression (Sauer et al., 2015; Khalid et al., 2016) show a reduction in gamma in cortical areas, hence being a putative endophenotype of depression (Fitzgerald and Watson, 2018). Most studies on gamma activity in depression and anxiety focused on the PFC. However, a study combining postmortem tissue and imaging from living depressed patients saw a dysfunction of inhibitory interneurons, considered to be responsible for gamma oscillations (Devalle et al., 2017), not only in the PFC and the ACC but also in the amygdala and hippocampus (Fee et al., 2017).

In the hippocampus, gamma oscillations correlate closely with information encoding during memory formation and its coupling with theta oscillation is necessary for hippocampus-neocortex communication (Lisman and Buzsáki, 2008). In the amygdala, gamma oscillations are generated both during the environment evaluation for assigning emotional value and spontaneously in rumination processes (Headley et al., 2021). Thus, the increase in gamma activity with FG-7142 in HPCv and BLA could reflect an increase in information processing to assign an emotional value.

However, the further increase in gamma activity in the entire circuit during DBS-IL is challenging to interpret. However, it might be associated with the therapeutic effect since gamma power increases in numerous effective anxiolytic and antidepressant treatments. In the application of transcranial magnetic stimulation, antidepressant success correlates with enhanced gamma signaling during and after recovery (Pathak et al., 2016; Noda et al., 2017; Bailey et al., 2018).

A similar effect occurs with ketamine antidepressant levels (Hakami et al., 2009; Hong et al., 2010; Gilbert and Zarate, 2020; Zacharias et al., 2020). Ketamine at subanesthetic doses has a rapid antidepressant action like the DBS (Matveychuk et al., 2020) and is also effective for some anxiety disorders, particularly for comorbid anxiety and depression (Ionescu et al., 2014). The increase in gamma power after subanesthetic administration of ketamine correlates with the antidepressant responses, and the power of this frequency band increased markedly in responders (Cornwell et al., 2012). Also depressed patients with lower basal gamma showed better response to treatment and a noticeable increase in gamma power in multiple brain regions. In contrast, control subjects had baseline higher gamma activity and did not exhibit clear responses to treatment. In general, the authors suggested that an optimal amount of gamma power in the circuits involved in depression could be associated with euthymia, that is, without mood alteration (Nugent et al., 2019).

With this in mind, a high basal gamma activity in female rats could correlate with a homeostatic protective mechanism (Gilbert and Zarate, 2020) modulating the activation and inhibition of the circuit, in this case, avoiding the alteration of the activity of the hippocampus and the amygdala.

Again, we have observed sexually differentiated connectivity patterns. In females, we found no relevant changes in the coupling study in the experimental conditions. However, in males, FG-7142 decreased communication at slow wave frequencies throughout the circuit and increased phase coupling and communication at delta and beta ranges between the ventral regions and at theta band between most regions. We could hypothesize that under anxiety conditions, the coordinated activity of the ventral subnetwork (HPCi-HPCv-BLA) communicates *via* delta, low theta, and beta. At the same time, intrahippocampal communication operates in the low theta range. The DBS-IL also reversed these patterns by recovering the synchronized activity at a slow frequency.

Many studies have linked the theta coupling in the amygdalo-hippocampal-prefrontal circuit with anxiety states and innate fear in rodents (Adhikari et al., 2010; Lesting et al., 2011; Seidenbecher and Lesting, 2012; Stujenske et al., 2014). Direct manipulation of theta interactions within this circuit produces a freezing state in rodents suggesting a causal relationship between these oscillations and states of fear and anxiety (Courtin et al., 2014; Padilla-Coreano et al., 2016). Our results in males coincide with previous works correlating this phenomenon with the pathophysiology of anxiety. A recent human study has reported a similar increase in WPLI at theta frequencies in patients suffering from generalized anxiety (Xing et al., 2016). Also, in a study using an experimental hyperanxiety model, the increase in theta coherence between HPCv and BLA was considerably increased during entry avoidance behaviors in the open arms of the elevated plus maze than in the closed arms (Jacinto et al., 2013).

In males, DBS-IL increased synchronization between slow-gamma and theta-gamma in the circuit. We previously showed these effects between HPCd and BLA in anesthetized animals without FG-7142 (Cervera-Ferri et al., 2016). The increase of theta-gamma coupling in males after the DBS-IL also coincides with work carried out in patients with treatment-resistant depression who underwent DBS applied in the anterior cingulate cortex (Sun et al., 2015). Other clinical studies indicate that the increase in theta-gamma coupling in the neocortex and the hippocampus was related to improving the mnemonic processes related to working memory (Canolty and Knight, 2010). Likewise, subanesthetic administration of ketamine also increases theta-gamma coupling in the hippocampus (Korotkova et al., 2010; Caixeta et al., 2013). However, females did not show any change in these parameters. Further analysis of these measures in females during behavioral and cognitive tests in anxiety models would be of interest to better understand the existing differences.

We have also observed a sexually differentiated pattern in the study of neuronal activation by c-Fos expression induced by FG-7142, with females showing a was much more restricted activation at telencephalic levels and only sharing with males in the diencephalic and brainstem levels studied. As expected, many areas with higher c-Fos expression in response to FG-7142, especially the amygdalar complex, the HPCv, the PFC, the septal area and the paraventricular nuclei, are strongly involved in anxiety processing. It is well established that CeA and BLA are involved, in humans and experimental models, in the facilitation of fear and anxiety, respectively, and their inhibition significantly reduces these behaviors (Charney et al., 1998; Ventura-Silva et al., 2013; Ranjbar et al., 2017). Our study in males is consistent with c-Fos expression in male rats in response to different anxiogenic conditions, including FG-7142 (Singewald et al., 2003; Funk et al., 2008).

In our study, FG-7142 administration in males increased c-Fos expression in HPCv and HPCd CA1 fields but not in the other hippocampal subregions. These results agree with several studies, also in male rodents, in which exposure to anxiety-inducing environments and FG-7142 increased expression levels of c-Fos in these regions (Kurumaji et al., 2003; Singewald et al., 2003; Funk et al., 2008). In contrast, we found no significant changes in the female group in the hippocampal formation or the amygdalar complex. This lack of activation confirms the electrophysiological study and is consistent with a recent work showing no significant variations in c-Fos expression in the hippocampus and amygdala of female rats after exposure to the elevated plus maze or the open field, regardless of the estrous phase (Wang et al., 2019).

At neocortical levels, the anxiogenic drug induced elevated c-Fos expression in IL in both sexes. Furthermore, in males, we observed a generalized increase in prefrontal activity. Overall, the hyperactivation observed in males in this work coincides with a previous study that also demonstrated a generalized activation pattern in these structures in response to FG-7142 (Salchner et al., 2006).

FG-714 also induced an increase in c-Fos expression in the LSI, the MS and the VDB. Several studies on the functionality of the septal area document that the injury and inhibition of the lateral and medial septum are anxiolytic (Trent and Menard, 2010; Chee et al., 2015; Parfitt et al., 2017). Also, its activation increases anxiety-related behaviors (Huang et al., 2021). The activation of the MS/VDB complex in males can explain the neuronal activation in the hippocampus and the generation of hippocampal theta since this structure is considered its primary pacemaker (Gaztelu and Buño, 1982). In females, the lack of activation is also consistent with the electrophysiological results.

On the other hand, the anxiogenic drug increased in both sexes neuronal activity in the paraventricular nuclei, the DR and the LC. These structures promote the release of hormones and neurotransmitters involved in neuroendocrine and autonomic responses that mediate fear behaviors and psychological stress (Lowry et al., 2008; Ryan et al., 2011; Rajkumar et al., 2016; Kirouac, 2021; Zeng et al., 2021). In general, our results in the diencephalon and brainstem match previous works. However, the activation of NI exclusively in males is striking, without changes in females. Lawther et al. (2018) documented its activation after the administration of FG-7142, but that work was done exclusively in males. The NI constitutes the primary input toward the MS/VDB complex (Teruel-Martí et al., 2008), which in turn projects toward the hippocampus. Thus, the activation of the NI exclusively in males could justify the different activation at the septohippocampal level and the lower presence of theta rhythm in females. Also, the NI projects directly to the amygdala and ventral hippocampus (Goto et al., 2001; Olucha-Bordonau et al., 2003; Smith et al., 2010), which could justify a more substantial influence on these regions than even on the HPCd.

Very similar increases had been described in male rats exposed to anxiogenic environmental situations (Singewald and Sharp, 2000; Singewald et al., 2003). Our results also agree with other works correlating anxiety responses in the elevated plus maze, conditioned fear or aversive conditioning with increased expression of c-Fos in the amygdalar complex (Saldívar-González et al., 2003; Sorregotti et al., 2018), the hippocampus (Galvis-Alonso et al., 2010), the prelimbic and infralimbic cortices (Duncan et al., 1996; Berg et al., 2019), the septal area (Reis et al., 2011; Chee et al., 2015), the diencephalon and brainstem (Singewald and Sharp, 2000; Hale et al., 2008; Rajkumar et al., 2016; Kirouac, 2021; Toropova et al., 2021; Zeng et al., 2021).

In our work, the lower neuronal activation in females, especially in the amygdala, hippocampus, and septal area, could be related to less expression of anxious behavior in response to FG-7142 (Meng and Drugan, 1993). Our results of neuronal activation in response to the combination of FG-7142 and DBS-IL indicate that its ability to change the activation pattern induced by the anxiogenic state could mediate part of its therapeutic action. In our case, 1 h of DBS-IL alone in both sexes already increased local activation in some regions. Other works have documented similar local increases in c-Fos expression after stimulation of the IL (Veerakumar et al., 2014) suggesting that high-frequency stimulation causes specific activation in the area surrounding the stimulation electrode. In addition, DBS-IL alone increased the activation in other cortical areas, most notably in males. A recent study found that DBS-IL increased the expression levels of zif268, another early expression gene used to study neuronal activation, in the prelimbic, cingulate, ectorrinal, orbital and temporal (Bregman et al., 2015). Since all these structures maintain direct or indirect connections with IL, our results support that the DBS not only acts locally. Instead, the high-frequency stimulation can also modulate the neuronal activity of structures connected to the DBS target region. However, the activation observed following the DBS-IL alone was always lower than with the anxiogenic drug.

Regarding the changes in the levels of neuronal activation induced by the combined application of DBS-IL and FG-7142, our results showed that acute stimulation counteracted the activation of all those regions activated by the anxiogenic drug. These data are consistent with the findings of studies that examined c-Fos expression in animals exposed to unpredictable chronic stress situations. While exposure to stressful stimuli increased c-Fos expression of DR-mediated serotonergic pathways, high-frequency stimulation significantly reduced that activation (Lim et al., 2015). It would indicate that the therapeutic action of DBS-IL applied in a pathological state can modulate the aberrant activity of those structures that connect with the stimulated region.

### 4.1. Limitations and future directions

With these considerations, the present study opens new research lines. On the one hand, it would be interesting to perform a detailed study of FG-7142 effects on females, and to investigate the behavioral correlates. Also, research is needed to understand the effects of DBS-IL in females, controlling estrous cycles or including ovariectomized females with controlled administration of sex hormones.

Our study also provides new data about sex differences in neuronal activation at the brainstem and telencephalic levels following the same dosage of an anxiogenic drug. Since this was the first study comparing brain oscillations using this drug in rats of both sexes, we used a single dose in males and females. Further studies should analyze whether different doses of the compound originate a similar response in females to that observed in males. Even, at basal anesthesia levels, there are differences in the oscillatory pattern that should be considered in electrophysiological studies.

## 5. Conclusion

FG-7142, a proven anxiogenic drug, originates a sexually differentiated response in the amygdalo-hippocampal network, which is key in emotional and cognitive processing. Surprisingly, the data point to a reduced response in female rats, contrasting with the higher prevalence of anxiety in women than in men. Also, our results show that DBS-IL can reverse aberrant oscillatory activity. Our study contributes to a better understanding of anxiety processing by the hippocampal formation and the amygdala, with a detailed study of the changes in the dorsoventral axis of the hippocampus, including the intermediate and ventral region, much less studied than the dorsal hippocampus. Also, our results point to the need to be careful in animal models’ translational studies of anxiety. Their different participation in pathological states and their therapeutic reversal offer new perspectives for future study.

## Data availability statement

The raw data supporting the conclusions of this article will be made available by the authors, without undue reservation.

## Ethics statement

All the experimental protocols were followed according to the Animal Care Guidelines of the European Communities Council Directive (2010/63/E.U.) and approved by the Research Ethics and Animal Welfare Committee of the University of Valencia (A20200227130134, A20191121151842, and A1537174325669) and the Valencian Government (Generalitat Valenciana) before performing the experiments.

## Author contributions

AC-F: conceptualization, project administration, supervision, and writing—original draft. HV-M and AG-M: data curation. HV-M: formal analysis. AC-F and AL: funding acquisition. HV-M, AG-M, RC-J, and AC-F: investigation. AB-S and AC-F: methodology. JM-R, AL, and AC-F: resources. VT-M, HV-M, AG-M, and AC-F: software. HV-M and AC-F: visualization. HV-M, AG-M, RC-J, JM-R, VT-M, AB-S, and AL: writing—review and editing. All authors have read and agreed to the published version of the manuscript.

## Supplementary material

Supplementary Figure 1 Statistical analysis of the peak frequency in females calculated by spectral decomposition. Please compare the results in females to that obtained in males [in Vila-Merkle et al. (2021)]. FG-7142 increases the peak frequency only in the HPCd and HPCi. However, group statistical results only show an increase to around 1.2 Hz. In the affected channels, the DBS restores the basal values. *Statistical significance in pairwise comparisons with the basal period. Triangle: statistical significance in pairwise comparisons between DBS-IL and post-DBS with the FG-7142 period (degree of significance not indicated to better visualize results; please see Table 1). **(C)** Comparative analysis between sexes. Neither the basal condition nor saline injection generated significant differences between sexes. However, the administration of FG-7142 and the application of the DBS-IL induces a differentiated and statistically significant frequency pattern between males and females. The asterisks denote the degree of significance in pairwise comparisons (^***^*p* < 0.001, ^**^*p* < 0.01, **p* < 0.05). BLA, basolateral amygdala; HPCd, dorsal hippocampus; HPCi, intermediate hippocampus; HPCv, ventral hippocampus.
