# Supplementary figures and images for: Sex differences in amygdalohippocampal oscillations and neuronal activation in a rodent anxiety model and in response to infralimbic deep brain stimulation
Source: Front Behav Neurosci. 2023 Feb 23;17:1122163. doi: 10.3389/fnbeh.2023.1122163 (PMC9995972; doi:10.3389/fnbeh.2023.1122163)

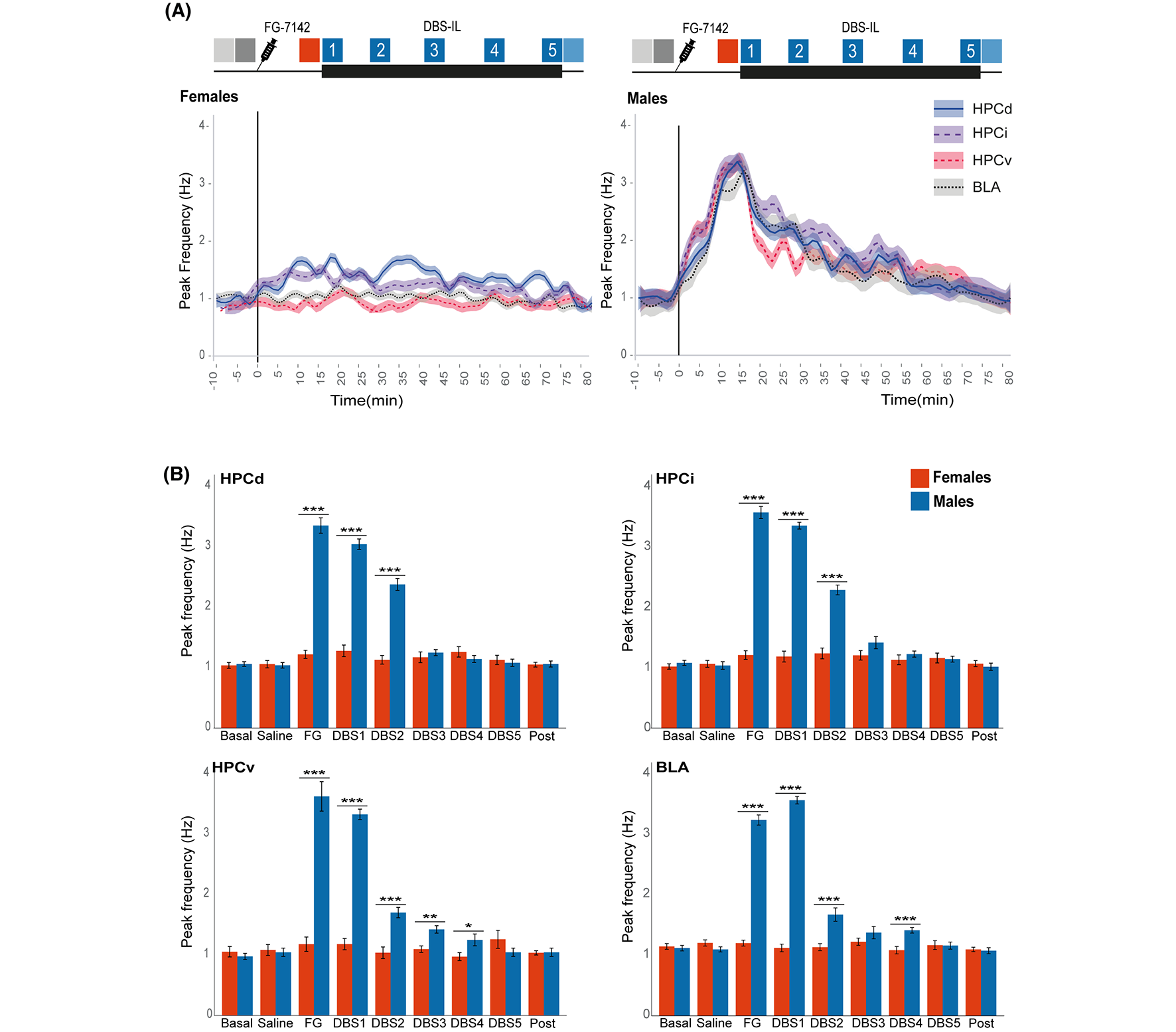

Supplement: Supplementary Figure 1 — Statistical analysis of the peak frequency in females calculated by spectral decomposition. Please compare the results in females to that obtained in males [in Vila-Merkle et al. (2021)]. FG-7142 increases the peak frequency only in the HPCd and HPCi. However, group statistical results only show an increase to around 1.2 Hz. In the affected channels, the DBS restores the basal values. *Statistical significance in pairwise comparisons with the basal period. Triangle: statistical significance in pairwise comparisons between DBS-IL and post-DBS with the FG-7142 period (degree of significance not indicated to better visualize results; please see Table 1). (C) Comparative analysis between sexes. Neither the basal condition nor saline injection generated significant differences between sexes. However, the administration of FG-7142 and the application of the DBS-IL induces a differentiated and statistically significant frequency pattern between males and females. The asterisks denote the degree of significance in pairwise comparisons (***p < 0.001, **p < 0.01, *p < 0.05). BLA, basolateral amygdala; HPCd, dorsal hippocampus; HPCi, intermediate hippocampus; HPCv, ventral hippocampus. [file Image_1.TIF]
