# Supplementary material for: Sex differences in amygdalohippocampal oscillations and neuronal activation in a rodent anxiety model and in response to infralimbic deep brain stimulation
Source: Front Behav Neurosci. 2023 Feb 23;17:1122163. doi: 10.3389/fnbeh.2023.1122163 (PMC9995972; doi:10.3389/fnbeh.2023.1122163)
Supplement: Supplementary file 2 [file Table_1.docx]

**Supplementary Table 1. Comparative analysis of the Relative Power of slow waves, delta, low theta, high theta and beta bands**

| **Band** | **Sex** | **Region** | **Basal** | **Saline** | **FG-7142** | **DBS1** | **DBS2** | **DBS3** | **DBS4** | **DBS5** | **POST-DBS** |
| --- | --- | --- | --- | --- | --- | --- | --- | --- | --- | --- | --- |
| **Slow Waves** | Male | **dHPC** | 0.49 ± 0.04 | 0.49 ± 0.04 | 0.28 ± 0.03 | **0.28 ± 0.04**** | 0.30 ± 0.04 | 0.34 ± 0.05 | 0.44 ± 0.05 | 0.45 ± 0.04 | 0.49 ± 0.04 |
|  | Female | **dHPC** | 0.46 ± 0.037 | 0.46 ± 0.04 | 0.36 ± 0.03 | **0.42 ± 0.04**** | 0.41 ± 0.04 | 0.42 ± 0.04 | 0.41 ± 0.04 | 0.42 ± 0.04 | 0.45 ± 0.03 |
|  | Male | **iHPC** | 0.38 ± 0.05 | 0.38 ± 0.05 | 0.22 ± 0.03 | 0.21 ± 0.04 | 0.22 ± 0.03 | 0.26 ± 0.05 | 0.35 ± 0.05 | 0.35 ± 0.05 | 0.40 ± 0.05 |
|  | Female | **iHPC** | 0.32 ± 0.03 | 0.32 ± 0.03 | 0.23 ± 0.02 | 0.28 ± 0.03 | 0.30 ± 0.04 | 0.31 ± 0.04 | 0.31 ± 0.05 | 0.32 ± 0.05 | 0.35 ± 0.05 |
|  | Male | **vHPC** | 0.44 ± 0.06 | 0.42 ± 0.07 | 0.23 ± 0.05 | 0.27 ± 0.05 | 0.26 ± 0.04 | 0.39 ± 0.08 | 0.39 ± 0.07 | 0.32 ± 0.06 | 0.37 ± 0.06 |
|  | Female | **vHPC** | 0.30 ± 0.03 | 0.31 ± 0.02 | 0.33 ± 0.02 | 0.32 ± 0.03 | 0.32 ± 0.03 | 0.33 ± 0.02 | 0.29 ± 0.03 | 0.30 ± 0.02 | 0.29 ± 0.04 |
|  | Male | **BLA** | 0.39 ± 0.04 | 0.40 ± 0.04 | **0.23 ± 0.04**** | **0.27 ± 0.05**** | **0.26 ± 0.04**** | 0.30 ± 0.05 | 0.35 ± 0.05 | 0.31 ± 0.04 | 0.38 ± 0.04 |
|  | Female | **BLA** | 0.44 ± 0.04 | 0.45 ± 0.04 | **0.42 ± 0.05**** | **0.40 ± 0.04**** | **0.43 ± 0.04**** | 0.40 ± 0.04 | 0.41 ± 0.04 | 0.42 ± 0.04 | 0.42 ± 0.03 |
| **Delta** | Male | **dHPC** | 0.16 ± 0.01 | 0.16 ± 0.01 | 0.16 ± 0.01 | 0.16 ± 0.02 | 0.16 ± 0.02 | 0.17 ± 0.02 | 0.16 ± 0.02 | 0.17 ± 0.01 | 0.17 ± 0.01 |
|  | Female | **dHPC** | 0.18 ± 0.01 | 0.18 ± 0.01 | 0.19 ± 0.01 | 0.18 ± 0.01 | 0.18 ± 0.01 | 0.20 ± 0.01 | 0.19 ± 0.01 | 0.18 ± 0.01 | 0.20 ± 0.01 |
|  | Male | **iHPC** | 0.15 ± 0.01 | 0.15 ± 0.01 | **0.25 ± 0.01**** | 0.13 ± 0.02 | 0.17 ± 0.02 | 0.16 ± 0.02 | 0.14 ± 0.02 | 0.14 ± 0.01 | 0.14 ± 0.01 |
|  | Female | **iHPC** | 0.17 ± 0.02 | 0.15 ± 0.01 | **0.18 ± 0.02**** | 0.16 ± 0.02 | 0.17 ± 0.02 | 0.17 ± 0.02 | 0.17 ± 0.02 | 0.15 ± 0.01 | 0.17 ± 0.01 |
|  | Male | **vHPC** | 0.17 ± 0.01 | 0.18 ± 0.02 | **0.27 ± 0.03**** | 0.21 ± 0.04 | 0.20 ± 0.02 | 0.16 ± 0.03 | 0.15 ± 0.03 | 0.16 ± 0.03 | 0.20 ± 0.02 |
|  | Female | **vHPC** | 0.14 ± 0.02 | 0.17 ± 0.02 | **0.14 ± 0.02**** | 0.16 ± 0.02 | 0.14 ± 0.02 | 0.16 ± 0.02 | 0.14 ± 0.02 | 0.19 ± 0.01 | 0.16 ± 0.02 |
|  | Male | **BLA** | 0.20 ± 0.01 | 0.20 ± 0.02 | **0.31 ± 0.03***** | 0.24 ± 0.04 | 0.21 ± 0.03 | 0.29 ± 0.03 | 0.18 ± 0.03 | 0.18 ± 0.03 | 0.19 ± 0.01 |
|  | Female | **BLA** | 0.16 ± 0.01 | 0.17 ± 0.01 | **0.17 ± 0.01***** | 0.17 ± 0.01 | 0.17 ± 0.01 | 0.19 ± 0.01 | 0.17 ± 0.01 | 0.17 ± 0.01 | 0.18 ± 0.01 |
| **Low Theta** | Male | **dHPC** | 0.19 ± 0.020 | 0.19 ± 0.02 | 0.32 ± 0.03* | 0.31 ± 0.03 | **0.32 ± 0.03*** | 0.29 ± 0.03 | 0.21 ± 0.03 | 0.20 ± 0.02 | 0.19 ± 0.02 |
|  | Female | **dHPC** | 0.20 ± 0.03 | 0.22 ± 0.03 | 0.27 ± 0.04 | 0.24 ± 0.03 | **0.24 ± 0.02*** | 0.23 ± 0.03 | 0.21 ± 0.03 | 0.20 ± 0.03 | 0.20 ± 0.02 |
|  | Male | **iHPC** | 0.20 ± 0.02 | 0.19 ± 0.02 | **0.30 ± 0.03*** | **0.34 ± 0.04***** | **0.32 ± 0.04**** | **0.31 ± 0.04**** | 0.22 ± 0.03 | 0.18 ± 0.02 | 0.17 ± 0.02 |
|  | Female | **iHPC** | 0.18 ± 0.02 | 0.17 ± 0.02 | **0.21 ± 0.03*** | **0.18 ± 0.02***** | **0.19 ± 0.02**** | **0.16 ± 0.02**** | 0.16 ± 0.03 | 0.18 ± 0.03 | 0.16 ± 0.02 |
|  | Male | **vHPC** | 0.16 ± 0.02 | 0.15 ± 0.02 | 0.23 ± 0.03 | 0.22 ± 0.03 | 0.23 ± 0.03 | 0.17 ± 0.03 | 0.18 ± 0.02 | 0.15 ± 0.02 | 0.17 ± 0.01 |
|  | Female | **vHPC** | 0.20 ± 0.02 | 0.21 ± 0.03 | 0.21 ± 0.04 | 0.19 ± 0.04 | 0.23 ± 0.04 | 0.20 ± 0.04 | 0.22 ± 0.04 | 0.21 ± 0.04 | 0.21 ± 0.03 |
|  | Male | **BLA** | 0.17 ± 0.02 | 0.15 ± 0.01 | **0.25 ± 0.03*** | 0.23 ± 0.03 | 0.22 ± 0.03 | 0.21 ± 0.03 | 0.19 ± 0.02 | 0.17 ± 0.02 | 0.16 ± 0.01 |
|  | Female | **BLA** | 0.20 ± 0.02 | 0.18 ± 0.02 | **0.16 ± 0.02*** | 0.18 ± 0.02 | 0.20 ± 0.02 | 0.19 ± 0.02 | 0.20 ± 0.02 | 0.17 ± 0.02 | 0.17 ± 0.02 |
| **High Theta** | Male | **dHPC** | 0.09 ± 0.01 | 0.08 ± 0.01 | **0.14 ± 0.01***** | **0.10 ± 0.01**** | 0.10 ± 0.01 | 0.08 ± 0.01 | 0.08 ± 0.01 | 0.08 ± 0.01 | 0.08 ± 0.01 |
|  | Female | **dHPC** | 0.08 ± 0.01 | 0.08 ± 0.01 | **0.08 ± 0.01***** | **0.07 ± 0.01**** | 0.08 ± 0.01 | 0.08 ± 0.01 | 0.08 ± 0.01 | 0.08 ± 0.01 | 0.07 ± 0.01 |
|  | Male | **iHPC** | 0.14 ± 0.03 | 0.16 ± 0.04 | **0.13 ± 0.01**** | **0.12 ± 0.01**** | 0.11 ± 0.01 | 0.12 ± 0.02 | 0.11 ± 0.01 | 0.11 ± 0.02 | 0.13 ± 0.02 |
|  | Female | **iHPC** | 0.11 ± 0.01 | 0.10 ± 0.01 | **0.09 ± 0.01**** | **0.09 ± 0.01**** | 0.09 ± 0.01 | 0.09 ± 0.01 | 0.08 ± 0.01 | 0.09 ± 0.01 | 0.08 ± 0.01 |
|  | Male | **vHPC** | 0.13 ± 0.03 | 0.16 ± 0.04 | 0.15 ± 0.02 | 0.12 ± 0.02 | 0.13 ± 0.02 | 0.10 ± 0.02 | 0.10 ± 0.02 | 0.12 ± 0.02 | 0.12 ± 0.02 |
|  | Female | **vHPC** | 0.12 ± 0.02 | 0.11 ± 0.01 | 0.11 ± 0.01 | 0.11 ± 0.02 | 0.10 ± 0.02 | 0.12 ± 0.02 | 0.10 ± 0.02 | 0.09 ± 0.01 | 0.11 ± 0.02 |
|  | Male | **BLA** | 0.16 ± 0.02 | 0.17 ± 0.03 | 0.16 ± 0.02 | 0.14 ± 0.02 | 0.13 ± 0.02 | 0.12 ± 0.02 | 0.11 ± 0.02 | 0.11 ± 0.02 | 0.13 ± 0.02 |
|  | Female | **BLA** | 0.12 ± 0.02 | 0.12 ± 0.02 | 0.12 ± 0.01 | 0.11 ± 0.01 | 0.11 ± 0.02 | 0.11 ± 0.01 | 0.10 ± 0.01 | 0.12 ± 0.02 | 0.10 ± 0.01 |
| **Beta** | Male | **dHPC** | 0.02 ± 4_*_10^-3^ | 0.02 ± 3_*_10^-4^ | 0.02 ± 4_*_10^-3^ | 0.02 ± 4_*_10^-3^ | 0.02 ± 4_*_10^-3^ | 0.02 ± 4_*_10^-3^ | 0.02 ± 4_*_10^-3^ | 0.02 ± 4_*_10^-3^ | 0.02 ± 0.04 |
|  | Female | **dHPC** | 0.02 ± 2_*_10^-3^ | 0.02 ± 2_*_10^-3^ | 0.02 ± 1_*_10^-3^ | 0.02 ± 1_*_10^-3^ | 0.02 ± 2_*_10^-3^ | 0.02 ± 2_*_10^-3^ | 0.02 ± 2_*_10^-3^ | 0.02 ± 2_*_10^-3^ | 0.02 ± 2_*_10^-3^ |
|  | Male | **iHPC** | 0.03 ± 4_*_10^-3^ | 0.03 ± 3_*_10^-4^ | **0.07 ± 0.01**** | 0.07 ± 0.01 | 0.06 ± 0.01 | 0.06 ± 0.01 | 0.04 ± 3_*_10^-3^ | 0.04 ± 3_*_10^-3^ | 0.04 ± 3_*_10^-3^ |
|  | Female | **iHPC** | 0.02 ± 2_*_10^-3^ | 0.02 ± 2_*_10^-3^ | **0.03 ± 2**_*_10^-3^****** | 0.03 ± 2_*_10^-3^ | 0.03 ± 2_*_10^-3^ | 0.02 ± 2_*_10^-3^ | 0.02 ± 2_*_10^-3^ | 0.02 ± 2_*_10^-3^ | 0.02 ± 2_*_10^-3^ |
|  | Male | **vHPC** | 0.02 ± 2_*_10^-3^ | 0.02 ± 1_*_10^-4^ | **0.07 ± 0.01***** | **0.05 ± 0.01**** | 0.05 ± 0.01 | 0.05 ± 0.01 | 0.04 ± 0.01 | 0.04 ± 3_*_10^-3^ | 0.03 ± 3_*_10^-3^ |
|  | Female | **vHPC** | 0.02 ± 3_*_10^-3^ | 0.02 ± 3_*_10^-3^ | **0.02 ± 3**_*_10^-3^******* | **0.02 ±3_*_10^-3^**** | 0.02 ± 2_*_10^-3^ | 0.02 ± 2_*_10^-3^ | 0.02 ± 2_*_10^-3^ | 0.02 ± 2_*_10^-3^ | 0.02 ± 3_*_10^-3^ |
|  | Male | **BLA** | 0.03 ± 3_*_10^-3^ | 0.03 ± 3_*_10^-4^ | **0.07 ± 0.01***** | **0.05 ± 0.01*** | 0.04 ± 0.01 | 0.04 ± 0.01 | 0.03 ± 0.01 | 0.03 ± 0.01 | 0.04 ± 3_*_10^-3^ |
|  | Female | **BLA** | 0.02 ± 1_*_10^-3^ | 0.02 ± 2_*_10^-4^ | **0.02 ± 2**_*_10^-3^******* | **0.02 ± 2_*_10^-3^*** | 0.02 ± 2_*_10^-3^ | 0.02 ± 2_*_10^-3^ | 0.02 ± 2_*_10^-3^ | 0.02 ± 2_*_10^-3^ | 0.02 ± 2_*_10^-3^ |

* Mean ± standard error. **Bold**: statistical significance between sexes; asterisks denote statistical significance between states ***p<0.001, **p<0.01, *p<0.05. BLA: basolateral amygdala; HPCd: dorsal hippocampus; HPCi: intermediate hippocampus; HPCv: ventral hippocampus.

±
