# Supplementary material for: Sex differences in amygdalohippocampal oscillations and neuronal activation in a rodent anxiety model and in response to infralimbic deep brain stimulation
Source: Front Behav Neurosci. 2023 Feb 23;17:1122163. doi: 10.3389/fnbeh.2023.1122163 (PMC9995972; doi:10.3389/fnbeh.2023.1122163)
Supplement: Supplementary file 3 [file Table_2.docx]

**­­Supplementary Table 2. Comparative analysis of the Gamma Logarithmic Power in both sexes**

| Band | Sex | Region | Basal | Saline | FG-7142 | DBS1 | DBS2 | DBS3 | DBS4 | DBS5 | POST-DBS |
| --- | --- | --- | --- | --- | --- | --- | --- | --- | --- | --- | --- |
| Low Gamma | Male | HPCd | **1.205 ± 0.024** | **1.196 ± 0.033** | 1.236 ± 0.026 | 1.590 ± 0.040 | 1.679 ± 0.066 | 1.684 ± 0.059 | 1.723 ± 0.064 | **1.651 ± 0.065** | 1.521 ± 0.056 |
|  | Female | HPCd | **1.504 ± 0.070*** | **1.493 ± 0.064**** | 1.373 ± 0.059 | 1.520 ± 0.059 | 1.498 ± 0.060 | 1.541 ± 0.065 | 1.510 ± 0.066 | **1.459 ± 0.071*** | 1.502 ± 0.068 |
|  | Male | HPCi | **2.208 ± 0.084** | **2.258 ± 0.080** | **2.570 ± 0.097** | **2.752 ± 0.100** | **2.780 ± 0.0.98** | **2.848 ± 0.101** | **2.939 ± 0.129** | **2.747 ± 0.078** | **2.596 ± 0.078** |
|  | Female | HPCi | **1.513 ± 0.054***** | **1.420 ± 0.060***** | **1.406 ± 0.062***** | **1.574 ± 0.056***** | **1.421 ± 0.059***** | **1.479 ± 0.067***** | **1.466 ± 0.060***** | **1.453 ± 0.057***** | **1.516 ± 0.055***** |
|  | Male | HPCv | 1.641 ± 0.060 | 1.609 ± 0.061 | 1.620 ± 0.055 | **2.007 ± 0.081** | **1.991 ± 0.095** | **1.989 ± 0.084** | **2.040 ± 0.081** | **2.000 ± 0.093** | **2.114 ± 0.081** |
|  | Female | HPCv | 1.514 ± 0.074 | 1.586 ± 0.069 | 1.659 ± 0.070 | **1.595 ± 0.076***** | **1.481 ± 0.088***** | **1.545 ± 0.075***** | **1.476 ± 0.071***** | **1.571 ± 0.068**** | **1.523 ± 0.075***** |
|  | Male | BLA | 1.332 ± 0.041 | 1.319 ± 0.047 | 1.357 ± 0.056 | 1.700 ± 0.074 | 1.761 ± 0.073 | 1.753 ± 0.065 | 1.732 ± 0.068 | **1.771 ± 0.059** | **1.719 ± 0.071** |
|  | Female | BLA | 1.475 ± 0.141 | 1.585 ± 0.065 | 1.425 ± 0.060 | 1.444± 0.065* | 1.552 ± 0.063 | 1.570 ± 0.066 | 1.528 ± 0.063 | **1.550 ± 0.066*** | **1.550 ± 0.080*** |
| Mid Gamma | Male | HPCd | **0.744 ± 0.047** | **0.737 ± 0.047** | **0.741 ± 0.041** | **1.247 ± 0.052** | **1.298 ± 0.045** | **1.340 ± 0.064** | **1.341 ± 0.064** | **1.338 ± 0.060** | 1.240 ± 0.072 |
|  | Female | HPCd | **1.571 ± 0.052***** | **1.567 ± 0.056***** | **1.538 ± 0.056***** | **1.518 ± 0.059**** | **1.591 ± 0.070***** | **1.538 ± 0.067*** | **1.571 ± 0.052**** | **1.511 ± 0.053*** | 4.318 ± 0.097 |
|  | Male | HPCi | **1.747 ± 0.075** | **1.768 ± 0.072** | **1.772 ± 0.073** | **1.737 ± 0.076** | **1.695 ± 0.073** | **1.698 ± 0.081** | **1.724 ± 0.080** | **1.712 ± 0.075** | 1.694 ± 0.080 |
|  | Female | HPCi | **1.486 ± 0.041*** | **1.541 ± 0.060 †** | **1.485 ± 0.058**** | **1.518 ± 0.062 †** | **1.431 ± 0.057**** | **1.421 ± 0.052*** | **1.406 ± 0.068**** | **1.436 ± 0.052*** | 3.774 ± 0.130 |
|  | Male | HPCv | **0.801 ± 0.046** | **0.822 ± 0.052** | **0.861 ± 0.050** | 1.494 ± 0.087 | 1.525 ± 0.089 | 1.501 ± 0.070 | 1.515 ± 0.070 | 1.456 ± 0.077 | 1.416 ± 0.077 |
|  | Female | HPCv | **1.531 ± 0.082***** | **1.590 ± 0.075***** | **1.498 ± 0.065***** | 1.409 ± 0.069 | 1.576 ± 0.054 | 1.557 ± 0.052 | 1.526 ± 0.067 | 1.520 ± 0.072 | 4.259 ± 0.199 |
|  | Male | BLA | **0.662 ± 0.048** | **0.676 ± 0.049** | **0.822 ± 0.060** | **1.079 ± 0.064** | **0.976 ± 0.056** | **1.084 ± 0.070** | **1.054 ± 0.070** | **1.088 ± 0.067** | 1.082 ± 0.073 |
|  | Female | BLA | **1.621 ± 0.052***** | **1.568 ± 0.051***** | **1.564 ± 0.062***** | **1.525 ± 0.058***** | **1.599 ± 0.053***** | **1.520 ± 0.054***** | **1.506 ± 0.053***** | **1.554 ± 0.052***** | 4.343 ± 0.112 |
| High Gamma | Male | HPCd | **0.598 ± 0.034** | **1.568 ± 0.051** | **0.596 ± 0.036** | **0.881 ± 0.075** | **0.843 ± 0.093** | **0.732 ± 0.049** | **0.801 ± 0.071** | **0.830 ± 0.082** | **0.711 ± 0.052** |
|  | Female | HPCd | **1.478 ± 0.048***** | **0.611 ± 0.033***** | **1.588 ± 0.059***** | **1.587 ± 0.055***** | **1.533 ± 0.063***** | **1.570 ± 0.064***** | **1.539 ± 0.072***** | **1.427 ± 0.065***** | **1.522 ± 0.067***** |
|  | Male | HPCi | **1.011 ± 0.066** | **1.500 ± 0.053** | **1.002 ± 0.078** | **1.308 ± 0.078** | **1.252 ± 0.070** | **1.255 ± 0.074** | **1.287 ± 0.063** | **1.215 ± 0.080** | **1.145 ± 0.078** |
|  | Female | HPCi | **1.623 ± 0.055***** | **1.039 ± 0.066***** | **1.553 ± 0.069***** | **1.519 ± 0.063***** | **1.529 ± 0.070***** | **1.454 ± 0.061***** | **1.461 ± 0.057***** | **1.578 ± 0.056***** | **1.511 ± 0.061***** |
|  | Male | HPCv | **0.523 ± 0.055** | **1.420 ± 0.057** | **0.803 ± 0.052** | **1.103 ± 0.089** | **1.145 ± 0.085** | **1.140 ± 0.071** | **1.104 ± 0.068** | 1.103 ± 0.059 | 1.131 ± 0.068 |
|  | Female | HPCv | **1.448 ± 0.070***** | **0.553 ± 0.043***** | **1.396 ± 0.077***** | **1.443 ± 0.082*** | **1.383 ± 0.079**** | **1.491 ± 0.090*** | **1.344 ± 0.086**** | 1.426 ± 0.07 | 1.371 ± 0.085 |
|  | Male | BLA | **0.576 ± 0.053** | **1.421 ± 0.075** | **0.554 ± 0.058** | **1.019 ± 0.052** | **0.813 ± 0.034** | **0.974 ± 0.054** | **0.906 ± 0.056** | **0.943 ± 0.054** | **0.790 ± 0.052** |
|  | Female | BLA | **1.521 ± 0.066***** | **0.583 ± 0.053***** | **1.471 ± 0.053***** | **1.541 ± 0.059***** | **1.394 ± 0.060***** | **1.420 ± 0.056***** | **1.463 ± 0.067***** | **1.390 ± 0.063***** | **1.444 ± 0.064***** |

Mean ± standard error. **Bold**: statistical significance between sexes; asterisks denote statistical significance between states ***p<0.001, **p<0.01, *p<0.05, cross: p<0.08. BLA: basolateral amygdala; HPCd: dorsal hippocampus; HPCi: intermediate hippocampus; HPCv: ventral hippocampus.

±
