# Supplementary material for: Sex differences in amygdalohippocampal oscillations and neuronal activation in a rodent anxiety model and in response to infralimbic deep brain stimulation
Source: Front Behav Neurosci. 2023 Feb 23;17:1122163. doi: 10.3389/fnbeh.2023.1122163 (PMC9995972; doi:10.3389/fnbeh.2023.1122163)
Supplement: Supplementary file 4 [file Table_3.docx]

**Supplementary Table 3. Comparative analysis of the Weighted Phase Lag Index in both sexes**

| **Band** | **Sex** | **Channels** | **Basal** | **Saline** | **FG-7142** | **DBS1** | **DBS2** | **DBS3** | **DBS4** | **DBS5** | **POST-DBS** |
| --- | --- | --- | --- | --- | --- | --- | --- | --- | --- | --- | --- |
| **Slow Waves** | Male | **dHPC-iHPC** | **0.890 ± 0.024** | **0.901 ± 0.018** | **0.260 ± 0.016** | **0.398 ± 0.030** | 0.645 ± 0.044 | 0.673 ± 0.042 | 0.669 ± 0.047 | 0.710 ± 0.031 | **0.888 ± 0.019** |
|  | Female | **dHPC-iHPC** | **0.589 ± 0.070**** | **0.593 ± 0.078*** | **0.620 ± 0.067***** | **0.676 ± 0.071***** | 0.609 ± 0.065 | 0.554 ± 0.079 | 0.500 ± 0.073 | 0.573 ± 0.067 | **0.707 ± 0.057*** |
|  | Male | **dHPC-BLA** | **0.869 ± 0.024** | **0.865 ± 0.021** | **0.350 ± 0.037** | 0.553 ± 0.041 | 0.555 ± 0.036 | 0.659 ± 0.047 | 0.686 ± 0.037 | 0.769 ± 0.020 | **0.825 ± 0.035** |
|  | Female | **dHPC-BLA** | **0.569 ± 0.073*** | **0.554 ± 0.067**** | **0.597 ± 0.069**** | 0.500 ± 0.077 | 0.677 ± 0.055 | 0.673 ± 0.061 | 0.644 ± 0.070 | 0.584 ± 0.079 | **0.605 ± 0.064*** |
|  | Male | **iHPC-BLA** | **0.896 ± 0.017** | **0.884 ± 0.033** | **0.253 ± 0.034** | 0.470 ± 0.031 | **0.459 ± 0.041** | 0.714 ± 0.031 | **0.834 ± 0.034** | **0.774 ± 0.032** | 0.861 ± 0.019 |
|  | Female | **iHPC-BLA** | **0.611 ± 0.072**** | **0.595 ± 0.073**** | **0.605 ± 0.070***** | 0.590 ± 0.076 | **0.695 ± 0.058**** | 0.704 ± 0.061 | **0.620 ± 0.069†** | **0.591 ± 0.069*** | 0.665 ± 0.068 |
|  | Male | **vHPC-dHPC** | 0.812 ± 0.034 | 0.801 ± 0.039 | **0.282 ± 0.033** | 0.419 ± 0.034 | **0.698 ± 0.063** | 0.787 ± 0.044 | 0.826 ± 0.042 | **0.779 ± 0.043** | **0.869 ± 0.028** |
|  | Female | **vHPC-dHPC** | 0.607 ± 0.095 | 0.565 ± 0.078 | **0.598 ± 0.073**** | 0.508 ± 0.091 | **0.496 ± 0.088 †** | 0.709 ± 0.078 | 0.631 ± 0.102 | **0.458 ± 0.097**** | **0.435 ± 0.081***** |
|  | Male | **vHPC-iHPC** | 0.807 ± 0.039 | 0.813 ± 0.025 | **0.255 ± 0.039** | **0.412 ± 0.047** | 0.579 ± 0.051 | 0.803 ± 0.039 | 0.795 ± 0.036 | 0.697 ± 0.037 | 0.845 ± 0.028 |
|  | Female | **vHPC-iHPC** | 0.812 ± 0.049 | 0.739 ± 0.059 | **0.725 ± 0.091***** | **0.686 ± 0.091*** | 0.682 ± 0.077 | 0.681 ± 0.095 | 0.618 ± 0.091 | 0.721 ± 0.090 | 0.602 ± 0.108 |
|  | Male | **vHPC-BLA** | 0.884 ± 0.019 | 0.889 ± 0.026 | **0.371 ± 0.034** | 0.749 ± 0.041 | 0.664 ± 0.048 | 0.747 ± 0.035^†^ | 0.911 ± 0.025 | 0.854 ± 0.034 | 0.849 ± 0.035 |
|  | Female | **vHPC-BLA** | 0.618 ± 0.104 | 0.649 ± 0.099 | **0.630 ± 0.093*** | 0.644 ± 0.113 | 0.705 ± 0.085 | 0.705 ± 0.105 | 0.833 ± 0.054 | 0.721 ± 0.099 | 0.673 ± 0.088 |
| **Delta** | Male | **dHPC-iHPC** | 0.414 ± 0.031 | 0.383 ± 0.033 | 0.428 ± 0.033 | 0.532 ± 0.045 | 0.582 ± 0.048 | 0.458 ± 0.046 | 0.556 ± 0.045 | **0.720 ± 0.046** | 0.540 ± 0.049 |
|  | Female | **dHPC-iHPC** | 0.541 ± 0.065 | 0.490 ± 0.071 | 0.515 ± 0.083 | 0.483 ± 0.061 | 0.540 ± 0.056 | 0.420 ± 0.058 | 0.490 ± 0.072 | **0.443 ± 0.074**** | 0.525 ± 0.065 |
|  | Male | **dHPC-BLA** | **0.583 ± 0.038** | **0.583 ± 0.044** | **0.652 ± 0.042** | **0.600 ± 0.048** | **0.596 ± 0.043** | **0.510 ± 0.043** | **0.607 ± 0.050** | **0.544 ± 0.054** | 0.497 ± 0.056 |
|  | Female | **dHPC-BLA** | **0.398 ± 0.054**** | **0.390 ± 0.057**** | **0.383 ± 0.044***** | **0.385 ± 0.049**** | **0.400 ± 0.037**** | **0.387 ± 0.050**** | **0.383 ± 0.050**** | **0.391 ± 0.044†** | 0.413 ± 0.040 |
|  | Male | **iHPC-BLA** | 0.337 ± 0.026 | 0.318 ± 0.022 | **0.796 ± 0.029** | **0.664 ± 0.046** | 0.484 ± 0.052 | 0.482 ± 0.048 | 0.493 ± 0.042 | 0.425 ± 0.032 | **0.328 ± 0.032** |
|  | Female | **iHPC-BLA** | 0.490 ± 0.06 | 0.470 ± 0.063 | **0.525 ± 0.069**** | **0.507 ± 0.063†** | 0.553 ± 0.058 | 0.521 ± 0.063 | 0.416 ± 0.059 | 0.418 ± 0.068 | **0.552 ± 0.067*** |
|  | Male | **vHPC-dHPC** | 0.542 ± 0.068 | 0.527 ± 0.065 | 0.453 ± 0.081 | 0.389 ± 0.080 | 0.405 ± 0.066 | 0.455 ± 0.080 | 0.411 ± 0.087 | 0.532 ± 0.080 | 0.438 ± 0.088 |
|  | Female | **vHPC-dHPC** | 0.425 ± 0.078 | 0.425 ± 0.039 | 0.440 ± 0.060 | 0.389 ± 0.050 | 0.452 ± 0.090 | 0.384 ± 0.040 | 0.467 ± 0.051 | 0.409 ± 0.036 | 0.490 ± 0.064 |
|  | Male | **vHPC-iHPC** | **0.325 ± 0.026** | **0.293 ± 0.037** | **0.780 ± 0.034** | 0.483 ± 0.042 | 0.604 ± 0.054 | **0.345 ± 0.043** | **0.349 ± 0.041** | **0.316 ± 0.026** | **0.326 ± 0.022** |
|  | Female | **vHPC-iHPC** | **0.625 ± 0.067**** | **0.592 ± 0.090*** | **0.562 ± 0.057**** | 0.563 ± 0.091 | 0.557 ± 0.078 | **0.573 ± 0.080*** | **0.535 ± 0.058*** | **0.525 ± 0.062**** | **0.555 ± 0.057**** |
|  | Male | **vHPC-BLA** | 0.367± 0.035 | 0.359± 0.051 | **0.811± 0.041** | 0.588± 0.067 | **0.761± 0.045** | 0.607± 0.066 | 0.705± 0.061 | 0.497± 0.083 | **0.213± 0.030** |
|  | Female | **vHPC-BLA** | 0.472± 0.080 | 0.498± 0.075 | **0.533± 0.075**** | 0.512± 0.092 | **0.552± 0.074†** | 0.520± 0.095 | 0.490± 0.098 | 0.500± 0.060 | **0.504± 0.074*** |
| **Low Theta** | Male | **dHPC-iHPC** | **0.218± 0.024** | **0.252± 0.026** | **0.771± 0.028** | **0.693± 0.039** | **0.689± 0.043** | **0.652± 0.045** | **0.714± 0.052** | 0.374± 0.043 | **0.266± 0.031** |
|  | Female | **dHPC-iHPC** | **0.477± 0.061**** | **0.471± 0.039***** | **0.476± 0.068**** | **0.490± 0.059*** | **0.477± 0.066*** | **0.489± 0.035**** | **0.470± 0.062**** | 0.491± 0.052 | **0.486± 0.057*** |
|  | Male | **dHPC-BLA** | 0.227± 0.027 | 0.282± 0.030 | **0.767± 0.028** | **0.524± 0.044** | **0.518± 0.038** | 0.366± 0.033 | 0.328± 0.037 | 0.247± 0.029 | **0.259± 0.037** |
|  | Female | **dHPC-BLA** | 0.304± 0.048 | 0.287± 0.058 | **0.306± 0.033***** | **0.323± 0.054**** | **0.285± 0.045***** | 0.315± 0.038 | 0.292± 0.039 | 0.301± 0.033 | **0.284± 0.022*** |
|  | Male | **iHPC-BLA** | 0.339± 0.040 | 0.334± 0.044 | **0.290± 0.045** | 0.328± 0.050 | 0.415± 0.060 | 0.320± 0.051 | 0.413± 0.057 | 0.348± 0.039 | 0.424± 0.062 |
|  | Female | **iHPC-BLA** | 0.350± 0.064 | 0.344± 0.062 | **0.390± 0.038*** | 0.356± 0.054 | 0.415± 0.059 | 0.334± 0.055 | 0.425± 0.059 | 0.343± 0.050 | 0.446± 0.062 |
|  | Male | **vHPC-dHPC** | **0.291± 0.033** | **0.257± 0.033** | **0.801± 0.032** | **0.613± 0.039** | 0.519± 0.044 | **0.591± 0.057** | **0.597± 0.071** | **0.331± 0.035** | **0.321± 0.033** |
|  | Female | **vHPC-dHPC** | **0.466± 0.078†** | **0.460± 0.038**** | **0.453± 0.051***** | **0.433± 0.038**** | 0.455± 0.049 | **0.399± 0.014**** | **0.396± 0.031*** | **0.438± 0.055*** | **0.443± 0.042**** |
|  | Male | **vHPC-iHPC** | **0.291± 0.016** | **0.285± 0.044** | **0.827± 0.042** | 0.482± 0.068 | **0.309± 0.054** | 0.497± 0.061 | 0.515± 0.061 | 0.343± 0.049 | 0.296± 0.023 |
|  | Female | **vHPC-iHPC** | **0.570± 0.046***** | **0.524± 0.084*** | **0.515± 0.047***** | 0.522± 0.082 | **0.521± 0.064*** | 0.503± 0.062 | 0.542± 0.055 | 0.490± 0.072 | 0.455± 0.085 |
|  | Male | **vHPC-BLA** | **0.239± 0.025** | **0.273± 0.022** | **0.788± 0.032** | 0.527± 0.061 | 0.475± 0.053 | 0.435± 0.061 | 0.382± 0.051 | **0.312± 0.033** | **0.213± 0.024** |
|  | Female | **vHPC-BLA** | **0.482± 0.078*** | **0.475± 0.083†** | **0.488± 0.065**** | 0.501± 0.052 | 0.460± 0.063 | 0.480± 0.047 | 0.484± 0.071 | **0.505± 0.077†** | **0.518± 0.065***** |
| **Beta** | Male | **dHPC-iHPC** | 0.091± 0.013 | 0.102± 0.012 | **0.097± 0.011** | **0.109± 0.012** | 0.139± 0.017 | **0.117± 0.015** | **0.124± 0.015** | **0.135± 0.017** | **0.128± 0.015** |
|  | Female | **dHPC-iHPC** | 0.284± 0.031 | 0.161± 0.024 | **0.198± 0.025**** | **0.203± 0.028**** | 0.201± 0.028 | **0.211± 0.036†** | **0.217± 0.033†** | **0.232± 0.026**** | **0.220± 0.031†** |
|  | Male | **dHPC-BLA** | 0.110± 0.014 | 0.108± 0.015 | 0.118± 0.013 | 0.131± 0.013 | **0.125± 0.012** | 0.133± 0.016 | 0.130± 0.015 | 0.141± 0.018 | 0.154± 0.014 |
|  | Female | **dHPC-BLA** | 0.151± 0.028 | 0.144± 0.024 | 0.155± 0.022 | 0.168± 0.027 | **0.155± 0.019†** | 0.124± 0.022 | 0.135± 0.028 | 0.165± 0.029 | 0.149± 0.023 |
|  | Male | **iHPC-BLA** | 0.168± 0.012 | 0.164± 0.014 | **0.419± 0.023** | **0.433± 0.027** | **0.343± 0.031** | **0.246± 0.017** | 0.158± 0.019 | 0.169± 0.014 | 0.167± 0.011 |
|  | Female | **iHPC-BLA** | 0.222± 0.032 | 0.225± 0.041 | **0.216± 0.026***** | **0.202± 0.037***** | **0.190± 0.019***** | **0.210± 0.021†** | 0.205± 0.023 | 0.294± 0.021 | 0.285± 0.033 |
|  | Male | **vHPC-dHPC** | 0.163± 0.018 | 0.135± 0.028 | 0.147± 0.027 | 0.127± 0.022 | 0.106± 0.016 | 0.159± 0.019 | 0.135± 0.024 | 0.138± 0.025 | **0.115± 0.028** |
|  | Female | **vHPC-dHPC** | 0.127± 0.029 | 0.115± 0.009 | 0.124± 0.032 | 0.126± 0.025 | 0.125± 0.034 | 0.164± 0.008 | 0.132± 0.016 | 0.150± 0.010 | **0.222± 0.011*** |
|  | Male | **vHPC-iHPC** | 0.111± 0.017 | 0.109± 0.019 | **0.411± 0.023** | **0.310± 0.029** | 0.291± 0.038 | **0.242± 0.027** | **0.353± 0.034** | **0.097± 0.023** | **0.115± 0.011** |
|  | Female | **vHPC-iHPC** | 0.188± 0.028 | 0.190± 0.046 | **0.187± 0.030***** | **0.188± 0.015**** | 0.194± 0.023 | **0.177± 0.020†** | **0.169± 0.016***** | **0.165± 0.025**** | **0.183± 0.026*** |
|  | Male | **vHPC-BLA** | **0.143± 0.022** | **0.146± 0.015** | **0.482± 0.018** | **0.280± 0.026** | 0.289± 0.041 | 0.233± 0.028 | 0.179± 0.030 | 0.177± 0.025 | 0.142± 0.021 |
|  | Female | **vHPC-BLA** | **0.222± 0.019*** | **0.222± 0.029*** | **0.197± 0.020***** | **0.206± 0.034†** | 0.206± 0.027 | 0.228± 0.040 | 0.229± 0.024 | 0.232± 0.025 | 0.217± 0.039 |

Mean ± standard error. **Bold**: statistical significance between sexes; asterisks denote statistical significance between states ***p<0.001, **p<0.01, *p<0.05. BLA: basolateral amygdala; HPCd: dorsal hippocampus; HPCi: intermediate hippocampus; HPCv: ventral hippocampus.

±
