# Supplementary material for: Sex differences in amygdalohippocampal oscillations and neuronal activation in a rodent anxiety model and in response to infralimbic deep brain stimulation
Source: Front Behav Neurosci. 2023 Feb 23;17:1122163. doi: 10.3389/fnbeh.2023.1122163 (PMC9995972; doi:10.3389/fnbeh.2023.1122163)
Supplement: Supplementary file 5 [file Table_4.docx]

Supplementary Table 4. Comparative analysis of Mutual Information in both sexes

| **Band** | **Sex** | **Channels** | **Basal** | Saline | **FG-7142** | **DBS1** | **DBS2** | **DBS3** | **DBS4** | **DBS5** | **POST-DBS** |
| --- | --- | --- | --- | --- | --- | --- | --- | --- | --- | --- | --- |
| **Slow Waves** | Male | **dHPC-iHPC** | 1.502 ± 0.065 | 1.491 ± 0.042 | **1.176 ± 1.051** | 1.372 ± 0.082 | 1.374 ± 0.078 | 1.487 ± 0.063 | 1.465 ± 0.046 | 1.522 ± 0.042 | 1.518 ± 0.054 |
|  | Female | **dHPC-iHPC** | 1.471 ± 0.114 | 1.525 ± 0.093 | **1.374 ± 0.094*** | 1.603 ± 0.119 | 1.350 ± 0.101 | 1.391 ± 0.078 | 1.359 ± 0.089 | 1.386 ± 0.103 | 1.339 ± 0.079 |
|  | Male | **dHPC-BLA** | 1.514 ± 0.061 | 1.505 ± 0.037 | **1.240 ± 0.030** | 1.444 ± 0.082 | 1.391 ± 0.074 | 1.447 ± 0.078 | 1.504 ± 0.065 | 1.477 ± 0.058 | 1.558 ± 0.067 |
|  | Female | **dHPC-BLA** | 1.462 ± 0.091 | 1.439 ± 0.082 | **1.356 ± 0.096 †** | 1.490 ± 0.117 | 1.332 ± 0.092 | 1.300 ± 0.097 | 1.277 ± 0.075 | 1.364 ± 0.098 | 1.324 ± 0.092 |
|  | Male | **iHPC-BLA** | **1.518 ± 0.067** | 1.505 ± 0.063 | 1.237 ± 0.044 | 1.288 ± 0.052 | **1.475 ± 0.079** | **1.520 ± 0.066** | **1.458 ± 0.054** | **1.485 ± 0.060** | **1.529 ± 0.067** |
|  | Female | **iHPC-BLA** | **1.326 ± 0.082*** | 1.473 ± 0.070 | 1.209 ± 0.073 | 1.436 ± 0.115 | **1.185 ± 0.070*** | **1.313 ± 0.097*** | **1.238 ± 0.067*** | **1.211 ± 0.068**** | **1.198 ± 0.061***** |
|  | Male | **vHPC-dHPC** | 1.604 ± 0.088 | 1.586 ± 0.080 | **1.230 ± 0.035** | 1.426 ± 0.081 | 1.376 ± 0.072 | 1.437 ± 0.123 | 1.495 ± 0.111 | 1.518 ± 0.115 | 1.588 ± 0.095 |
|  | Female | **vHPC-dHPC** | 1.409 ± 0.081 | 1.568 ± 0.107 | **1.522 ± 0.126*** | 1.558 ± 0.174 | 1.435 ± 0.112 | 1.433 ± 0.112 | 1.405 ± 0.138 | 1.430 ± 0.159 | 1.393 ± 0.101 |
|  | Male | **vHPC-iHPC** | 1.519 ± 0.106 | 1.511 ± 0.086 | **1.187 ± 0.030** | 1.418 ± 0.080 | 1.461 ± 0.129 | 1.385 ± 0.106 | 1.436 ± 0.122 | 1.499 ± 0.079 | 1.577 ± 0.103 |
|  | Female | **vHPC-iHPC** | 1.384 ± 0.105 | 1.548 ± 0.104 | **1.419 ± 0.109*** | 1.493 ± 0.157 | 1.338 ± 0.103 | 1.430 ± 0.115 | 1.365 ± 0.110 | 1.395 ± 0.126 | 1.414 ± 0.061 |
|  | Male | **vHPC-BLA** | 1.622 ± 0.055 | 1.644 ± 0.076 | **1.231 ± 0.027** | 1.443 ± 0.058 | 1.488 ± 0.088 | 1.588 ± 0.097 | 1.580 ± 0.088 | 1.627 ± 0.092 | 1.656 ± 0.053 |
|  | Female | **vHPC-BLA** | 1.630 ± 0.117 | 1.747 ± 0.105 | **1.637 ± 0.143*** | 1.751 ± 0.149 | 1.595 ± 0.157 | 1.564 ± 0.135 | 1.438 ± 0.123 | 1.569 ± 0.152 | 1.498 ± 0.138 |
| **Delta** | Male | **dHPC-iHPC** | 0.963 ± 0.051 | **0.986 ± 0.059** | **0.930 ± 0.072 *** | 0.978 ± 0.063 | 0.938 ± 0.060 | 0.924 ± 0.059 | 1.021 ± 0.056 | 0.943 ± 0.050 | 0.981 ± 0.059 |
|  | Female | **dHPC-iHPC** | 1.072 ± 0.081 | **1.224 ± 0.078*** | **0.995 ± 0.070*** | 0.996 ± 0.078 | 0.979 ± 0.063 | 1.115 ± 0.069 | 1.025 ± 0.062 | 0.960 ± 0.071 | 1.047 ± 0.088 |
|  | Male | **dHPC-BLA** | 1.016 ± 0.043 | 1.025 ± 0.044 | **1.045 ± 0.056 *** | 1.066 ± 0.049 | 1.049 ± 0.047 | 1.003 ± 0.047 | 1.097 ± 0.045 | 1.040 ± 0.041 | 1.028 ± 0.046 |
|  | Female | **dHPC-BLA** | 1.117 ± 0.073 | 1.057 ± 0.081 | **1.015 ± 0.078** | 1.060 ± 0.104 | 0.976 ± 0.090 | 1.058 ± 0.073 | 1.017 ± 0.079 | 1.035 ± 0.080 | 1.032 ± 0.106 |
|  | Male | **iHPC-BLA** | 0.986 ± 0.049 | 1.043 ± 0.054 | 1.019 ± 0.084 | 1.028 ± 0.074 | 0.987 ± 0.066 | 0.917 ± 0.048 | 0.999 ± 0.057 | 0.937 ± 0.053 | 0.952 ± 0.053 |
|  | Female | **iHPC-BLA** | 0.971 ± 0.065 | 1.117 ± 0.066 | 0.904 ± 0.053 | 0.911 ± 0.058 | 0.911 ± 0.062 | 0.970 ± 0.045 | 0.954 ± 0.053 | 0.893 ± 0.051 | 0.991 ± 0.068 |
|  | Male | **vHPC-dHPC** | 1.017 ± 0.072 | 1.110 ± 0.080 | 1.071 ± 0.072 | 1.050 ± 0.060 | 1.044 ± 0.078 | 1.069 ± 0.095 | 1.026 ± 0.075 | 1.023 ± 0.067 | 1.065 ± 0.082 |
|  | Female | **vHPC-dHPC** | 1.203 ± 0.098 | 1.295 ± 0.082 | 1.082 ± 0.094 | 1.027 ± 0.087 | 1.073 ± 0.089 | 1.133 ± 0.097 | 1.130 ± 0.123 | 1.036 ± 0.087 | 1.025 ± 0.081 |
|  | Male | **vHPC-iHPC** | 1.065 ± 0.101 | 1.053 ± 0.056 | 1.335 ± 0.039 | 1.113 ± 0.089 | 1.141 ± 0.099 | 1.075 ± 0.100 | 0.980 ± 0.065 | 1.021 ± 0.069 | 1.060 ± 0.064 |
|  | Female | **vHPC-iHPC** | 1.193 ± 0.097 | 1.284 ± 0.101 | 1.162 ± 0.086 | 1.054 ± 0.113 | 1.079 ± 0.095 | 1.147 ± 0.102 | 0.223 ± 0.107 | 1.114 ± 0.110 | 1.178 ± 0.075 |
|  | Male | **vHPC-BLA** | 1.183 ± 0.085 | 1.181 ± 0.066 | 1.515 ± 0.075 | 1.443 ± 0.088 | 1.417 ± 0.115 | 1.225 ± 0.083 | 1.178 ± 0.053 | 1.199 ± 0.083 | 1.212 ± 0.086 |
|  | Female | **vHPC-BLA** | 1.345 ± 0.122 | 1.375 ± 0.114 | 1.294 ± 0.114 | 1.293 ± 0.132 | 1.238 ± 0.147 | 1.335 ± 0.118 | 1.263 ± 0.118 | 1.365 ± 0.097 | 1.305 ± 0.129 |
| **Low Theta** | Male | **dHPC-iHPC** | **0.623 ± 0.019** | **0.611 ± 0.030** | 0.825 ± 0.041 | 0.816 ± 0.033 | 0.730 ± 0.038 | 0.745 ± 0.032 | **0.667 ± 0.042** | **0.621 ± 0.037** | **0.637 ± 0.036** |
|  | Female | **dHPC-iHPC** | **0.928 ± 0.065***** | **0.962 ± 0.067***** | 0.769 ± 0.074 | 0.824 ± 0.080 | 0.778 ± 0.074 | 0.841 ± 0.072 | **0.857 ± 0.074 †** | **0.829 ± 0.068*** | **0.849 ± 0.062*** |
|  | Male | **dHPC-BLA** | 0.804 ± 0.044 | 0.795 ± 0.033 | 0.786 ± 0.038 | 0.813 ± 0.045 | 0.794 ± 0.044 | 0.720 ± 0.034 | 0.789 ± 0.043 | 0.773 ± 0.048 | 0.786 ± 0.051 |
|  | Female | **dHPC-BLA** | 0.920 ± 0.067 | 0.946 ± 0.063 | 0.906 ± 0.056 | 0.895 ± 0.075 | 0.884 ± 0.053 | 0.848 ± 0.066 | 0.906 ± 0.076 | 0.828 ± 0.046 | 0.863 ± 0.076 |
|  | Male | **iHPC-BLA** | 0.739 ± 0.045 | 0.736 ± 0.044 | 0.749 ± 0.064 | 0.744 ± 0.066 | 0.721 ± 0.052 | 0.737 ± 0.032 | 0.717 ± 0.055 | 0.739 ± 0.041 | 0.722 ± 0.042 |
|  | Female | **iHPC-BLA** | 0.899 ± 0.073 | 0.890 ± 0.074 | 0.740 ± 0.065 | 0.802 ± 0.054 | 0.777 ± 0.051 | 0.777 ± 0.071 | 0.761 ± 0.064 | 0.767 ± 0.060 | 0.790 ± 0.064 |
|  | Male | **vHPC-dHPC** | 0.651 ± 0.040 | 0.676 ± 0.042 | 0.856 ± 0.035 | 0.816 ± 0.070 | 0.731 ± 0.064 | 0.705 ± 0.073 | 0.700 ± 0.061 | 0.677 ± 0.072 | 0.657 ± 0.038 |
|  | Female | **vHPC-dHPC** | 0.057 ± 0.087 | 1.003 ± 0.063 | 0.892 ± 0.089 | 0.886 ± 0.093 | 0.906 ± 0.074 | 0.945 ± 0.078 | 0.894 ± 0.076 | 0.868 ± 0.058 | 0.958 ± 0.070 |
|  | Male | **vHPC-iHPC** | **0.595 ± 0.014** | **0.608 ± 0.013** | 0.875 ± 0.049 | 0.834 ± 0.091 | 0.749 ± 0.086 | 0.720 ± 0.054 | 0.717 ± 0.055 | **0.661 ± 0.054** | **0.668 ± 0.031** |
|  | Female | **vHPC-iHPC** | **0.935 ± 0.101*** | **0.995 ± 0.089**** | 0.855 ± 0.104 | 0.758 ± 0.100 | 0.779 ± 0.097 | 0.850 ± 0.098 | 0.798 ± 0.100 | **0.830 ± 0.095 †** | **0.891 ± 0.089**** |
|  | Male | **vHPC-BLA** | **0.873 ± 0.095** | **0.850 ± 0.093** | **0.908 ± 0.065** | **0.915 ± 0.093** | **0.928 ± 0.107** | **0.703 ± 0.106** | **0.790 ± 0.074** | **0.784 ± 0.090** | **0.841 ± 0.079** |
|  | Female | **vHPC-BLA** | **1.226 ± 0.140 †** | **1.221 ± 0.122*** | **1.132 ± 0.096*** | **1.218 ± 0.099 †** | **1.129 ± 0.119*** | **1.157 ± 0.113**** | **1.084 ± 0.103*** | **1.178 ± 0.133*** | **1.141 ± 0.112 †** |
| **Beta** | Male | **dHPC-iHPC** | 0.386 ± 0.031 | 0.387 ± 0.022 | **0.384 ± 0.025** | **0.387 ± 0.028** | **0.378 ± 0.028** | **0.388 ± 0.036** | **0.390 ± 0.029** | 0.385 ± 0.026 | 0.374 ± 0.031 |
|  | Female | **dHPC-iHPC** | 0.370 ± 0.028 | 0.340 ± 0.019 | **0.311 ± 0.016 †** | **0.320 ± 0.024*** | **0.298 ± 0.015**** | **0.320 ± 0.024**** | **0.337 ± 0.034*** | 0.367 ± 0.042 | 0.362 ± 0.038 |
|  | Male | **dHPC-BLA** | 0.507 ± 0.042 | 0.502 ± 0.039 | **0.480 ± 0.040** | **0.509 ± 0.042** | **0.494 ± 0.043** | **0.455 ± 0.031** | **0.483 ± 0.033** | **0.478 ± 0.035** | 0.483 ± 0.038 |
|  | Female | **dHPC-BLA** | 0.423 ± 0.038 | 0.436 ± 0.033 | **0.346 ± 0.021*** | **0.419 ± 0.042 †** | **0.351 ± 0.025**** | **0.360 ± 0.029**** | **0.380 ± 0.034*** | **0.411 ± 0.041 †** | 0.401 ± 0.028 |
|  | Male | **iHPC-BLA** | 0.471 ± 0.040 | 0.465 ± 0.037 | 0.431 ± 0.039 | 0.478 ± 0.046 | **0.440 ± 0.036** | 0.437 ± 0.035 | 0.433 ± 0.033 | 0.421 ± 0.031 | 0.424 ± 0.027 |
|  | Female | **iHPC-BLA** | 0.450 ± 0.040 | 0.432 ± 0.041 | 0.348 ± 0.025 | 0.403 ± 0.040 | **0.372 ± 0.039 †** | 0.373 ± 0.035 | 0.424 ± 0.060 | 0.443 ± 0.062 | 0.459 ± 0.054 |
|  | Male | **vHPC-dHPC** | 0.480 ± 0.045 | 0.469 ± 0.050 | **0.447 ± 0.032** | **0.500 ± 0.042** | **0.445 ± 0.032** | **0.422 ± 0.035** | **0.411 ± 0.036** | 0.404 ± 0.028 | 0.401 ± 0.036 |
|  | Female | **vHPC-dHPC** | 0.434 ± 0.061 | 0.431 ± 0.060 | **0.415 ± 0.072*** | **0.419 ± 0.059*** | **0.383 ± 0.056*** | **0.400 ± 0.079*** | **0.394 ± 0.070 †** | 0.398 ± 0.069 | 0.374 ± 0.049 |
|  | Male | **vHPC-iHPC** | 0.424 ± 0.019 | 0.418 ± 0.033 | **0.612 ± 0.028** | **0.557 ± 0.064** | **0.528 ± 0.058** | **0.461 ± 0.032** | 0.406 ± 0.047 | 0.429 ± 0.043 | 0.442 ± 0.052 |
|  | Female | **vHPC-iHPC** | 0.410 ± 0.042 | 0.413 ± 0.038 | **0.241 ± 0.023***** | **0.367 ± 0.030*** | **0.355 ± 0.033**** | **0.367 ± 0.030*** | 0.406 ± 0.064 | 0.445 ± 0.079 | 0.425 ± 0.067 |
|  | Male | **vHPC-BLA** | 0.520 ± 0.058 | 0.523 ± 0.071 | 0.870 ± 0.050 | 0.813 ± 0.070 | 0.666 ± 0.083 | 0.653 ± 0.103 | 0.631 ± 0.087 | 0.480 ± 0.050 | 0.515 ± 0.062 |
|  | Female | **vHPC-BLA** | 0.685 ± 0.099 | 0.675 ± 0.094 | 0.571 ± 0.112 | 0.652 ± 0.105 | 0.578 ± 0.102 | 0.568 ± 0.103 | 0.580 ± 0.112 | 0.585 ± 0.099 | 0.609 ± 0.097 |

* Mean ± standard error. **Bold**: statistical significance between sexes; asterisks denote statistical significance between states ***p<0.001, **p<0.01, *p<0.05, cross: p<0.08. BLA: basolateral amygdala; HPCd: dorsal hippocampus; HPCi: intermediate hippocampus; HPCv: ventral hippocampus.

±
