# Supplementary material for: Sex differences in amygdalohippocampal oscillations and neuronal activation in a rodent anxiety model and in response to infralimbic deep brain stimulation
Source: Front Behav Neurosci. 2023 Feb 23;17:1122163. doi: 10.3389/fnbeh.2023.1122163 (PMC9995972; doi:10.3389/fnbeh.2023.1122163)
Supplement: Supplementary file 6 [file Table_5.docx]

**Supplementary Table 5. Comparative analysis of the Phase-Amplitude Coupling in both sexes**

| **Bands** | **Sex** | **Region** | **Basal** | **Saline** | **FG-7142** | **DBS1** | **DBS2** | **DBS3** | **DBS4** | **DBS5** | **POST-DBS** |
| --- | --- | --- | --- | --- | --- | --- | --- | --- | --- | --- | --- |
| **Slow Waves-**  **Low Gamma** | Male | **dHPC** | 0.0013 ± 0.0002 | 0.0012 ± 0.0002 | 0.0011 ± 0.0002 | **0.0035 ± 0.0004** | **0.0016 ± 0.0001** | **0.0023 ± 0.0001** | **0.0024 ± 0.0002** | **0.0026 ± 0.0002** | **0.0027 ± 0.0002** |
|  | Female | **dHPC** | 0.0015 ± 0.0002 | 0.00152 ± 0.00022 | 0.00148 ± 0.00020 | **0.00156 ± 0.00018***** | **0.00137 ± 0.00015*** | **0.00161 ± 0.00009***** | **0.00146 ± 0.00013***** | **0.00147 ± 0.00015***** | **0.00152 ± 0.00022***** |
|  | Male | **iHPC** | **0.0039 ± 0.0002** | **0.0038 ± 0.0003** | **0.0039 ± 0.0004** | **0.0066 ± 0.0005** | **0.0056 ± 0.0004** | **0.0058 ± 0.0005** | **0.0056 ± 0.0005** | **0.0066 ± 0.0005** | **0.0062 ± 0.0005** |
|  | Female | **iHPC** | **0.0020 ± 0.0002***** | **0.00176 ± 0.00016***** | **0.00182 ± 0.00026***** | **0.00178 ± 0.00015***** | **0.00182 ± 0.00024***** | **0.00181 ± 0.00021***** | **0.00182 ± 0.00015***** | **0.00180 ± 0.00022***** | **0.00180 ± 0.00022***** |
|  | Male | **vHPC** | 0.0014 ± 0.0001 | 0.0014 ± 0.0002 | 0.0016 ± 0.0002 | **0.0034 ± 0.0003** | **0.0044 ± 0.0004** | **0.0032 ± 0.0003** | **0.0026 ± 0.0004** | **0.0028 ± 0.0004** | **0.0029 ± 0.0004** |
|  | Female | **vHPC** | 0.0012 ± 0.0003 | 0.00127 ± 0.00026 | 0.00130 ± 0.00021 | **0.00115 ± 0.00018***** | **0.00145 ± 0.00021***** | **0.00122 ± 0.00019***** | **0.00143 ± 0.00019***** | **0.00121 ± 0.00016***** | **0.00128 ± 0.00035***** |
|  | Male | **BLA** | 0.0012 ± 0.0001 | 0.0012 ± 0.0001 | 0.0011 ± 0.0001 | **0.0035 ± 0.0002** | **0.0025 ± 0.0004** | **0.0024 ± 0.0003** | **0.0024 ± 0.0003** | **0.0022 ± 0.0002** | **0.0036 ± 0.0003** |
|  | Female | **BLA** | 0.0011 ± 0.0002 | 0.00126 ± 0.00029 | 0.00127 ± 0.00022 | **0.00139 ± 0.00016***** | **0.00131 ± 0.00010***** | **0.00145 ± 0.00018***** | **0.00129 ± 0.00018***** | **0.00132 ± 0.00020***** | **0.00133 ± 0.00019***** |
| **Delta- Beta** | Male | **dHPC** | 0.0012 ± 0.0001 | 0.0011 ± 0.0001 | 0.0011 ± 0.0001 | **0.0028 ± 0.0002** | **0.0022 ± 0.0002** | **0.0022 ± 0.0002** | **0.0022 ± 0.0002** | **0.0021 ± 0.0002** | **0.0024 ± 0.0002** |
|  | Female | **dHPC** | 0.0013 ± 0.0001 | 0.00132 ± 0.00026 | 0.00137 ± 0.00026 | **0.00130 ± 0.00016***** | **0.00126 ± 0.00021***** | **0.00127 ± 0.00020**** | **0.00131 ± 0.00019***** | **0.00124 ± 0.00025***** | **0.00129 ± 0.00025***** |
|  | Male | **iHPC** | 0.0032 ± 0.0003 | 0.0028 ± 0.0001 | **0.0076 ± 0.0009** | **0.0041 ± 0.0003** | **0.0044 ± 0.0004** | **0.0034 ± 0.0003** | **0.0031 ± 0.0003** | **0.0035 ± 0.0004** | **0.0032 ± 0.0003** |
|  | Female | **iHPC** | 0.0024 ± 0.0003 | 0.00234 ± 0.00040 | **0.00213 ± 0.00042***** | **0.00226 ± 0.00038**** | **0.00217 ± 0.00038***** | **0.00226 ± 0.00038**** | **0.00197 ± 0.00025**** | **0.00225 ± 0.00037**** | **0.00228 ± 0.00030*** |
|  | Male | **vHPC** | 0.0013 ± 0.0001 | 0.0013 ± 0.0002 | **0.0035 ± 0.0003** | **0.0037 ± 0.0004** | **0.0025 ± 0.0001** | 0.0020 ± 0.0002 | **0.0027 ± 0.0002** | 0.0021 ± 0.0002 | 0.0016 ± 0.0002 |
|  | Female | **vHPC** | 0.0019 ± 0.0004 | 0.00183 ± 0.00024 | **0.00167 ± 0.00023***** | **0.00193 ± 0.00040**** | **0.00185 ± 0.00035**** | 0.00188 ± 0.00017 | **0.00200 ± 0.00035*** | 0.00183 ± 0.00017 | 0.00188 ± 0.00017 |
|  | Male | **BLA** | **0.0028 ± 0.0002** | **0.0027 ± 0.0003** | **0.0043 ± 0.0003** | **0.0031 ± 0.0002** | **0.0024 ± 0.0002** | **0.0023 ± 0.0002** | 0.0020 ± 0.0002 | **0.0022 ± 0.0003** | 0.0021 ± 0.0002 |
|  | Female | **BLA** | **0.0019 ± 0.0004**** | **0.00190 ± 0.00033*** | **0.00184 ± 0.00038***** | **0.00176 ± 0.00046***** | **0.00166 ± 0.00039**** | **0.00167 ± 0.00031*** | 0.00186 ± 0.00034 | **0.00171 ± 0.00039*** | 0.00169 ± 0.00020 |
| **Low Theta –**  **Low Gamma** | Male | **dHPC** | 0.0004 ± 0.0001 | 0.0004 ± 0.0000 | 0.0004 ± 0.0000 | **0.0018 ± 0.0002** | **0.0017 ± 0.0003** | **0.0010 ± 0.0001** | **0.0010 ± 0.0001** | **0.0010 ± 0.0001** | **0.0012 ± 0.0001** |
|  | Female | **dHPC** | 0.0006 ± 0.0001 | 0.00132 ± 0.00026 | 0.00137 ± 0.00026 | **0.00130 ± 0.00016***** | **0.00126 ± 0.00021***** | **0.00127 ± 0.00020***** | **0.00131 ± 0.00019**** | **0.00124 ± 0.00025**** | **0.00129 ± 0.00025***** |
|  | Male | **iHPC** | **0.0012 ± 0.0001** | **0.0012 ± 0.0001** | **0.0016 ± 0.0001** | **0.0025 ± 0.0002** | **0.0023 ± 0.0002** | **0.0035 ± 0.0002** | **0.0025 ± 0.0002** | **0.0032 ± 0.0003** | **0.0032 ± 0.0002** |
|  | Female | **iHPC** | **0.0008 ± 0.0001**** | **0.00234 ± 0.00040**** | **0.00213 ± 0.00042***** | **0.00226 ± 0.00038***** | **0.00217 ± 0.00038***** | **0.00226 ± 0.00038***** | **0.00197 ± 0.00025***** | **0.00225 ± 0.00037***** | **0.00228 ± 0.00030***** |
|  | Male | **vHPC** | 0.0007 ± 0.0001 | 0.0007 ± 0.0002 | 0.0008 ± 0.0002 | **0.0020 ± 0.0002** | **0.0015 ± 0.0002** | **0.0017 ± 0.0002** | **0.0013 ± 0.0001** | **0.0014 ± 0.0002** | **0.0015 ± 0.0002** |
|  | Female | **vHPC** | 0.0005 ± 0.0001 | 0.00183 ± 0.00024 | 0.00167 ± 0.00023 | **0.00193 ± 0.00040***** | **0.00185 ± 0.00035***** | **0.00188 ± 0.00017***** | **0.00200 ± 0.00035***** | **0.00183 ± 0.00017***** | **0.00188 ± 0.00017***** |
|  | Male | **BLA** | 0.0004 ± 0.0001 | 0.0004 ± 0.0000 | 0.0004 ± 0.0000 | 0.0017 ± 0.0002 | 0.0011 ± 0.0001 | **0.0009 ± 0.0001** | **0.0008 ± 0.0001** | **0.0011 ± 0.0001** | **0.0012 ± 0.0001** |
|  | Female | **BLA** | 0.0005 ± 0.0001 | 0.00190 ± 0.00033 | 0.00184 ± 0.00038 | 0.00176 ± 0.00046 | 0.00166 ± 0.00039 | **0.00167 ± 0.00031***** | **0.00186 ± 0.00034***** | **0.00171 ± 0.00039***** | **0.00169 ± 0.00020***** |

Mean ± standard error. **Bold**: statistical significance between sexes; asterisks denote statistical significance between states ***p<0.001, **p<0.01, *p<0.05. BLA: basolateral amygdala; HPCd: dorsal hippocampus; HPCi: intermediate hippocampus; HPCv: ventral hippocampus.

±
