# Supplementary material for: Sex differences in amygdalohippocampal oscillations and neuronal activation in a rodent anxiety model and in response to infralimbic deep brain stimulation
Source: Front Behav Neurosci. 2023 Feb 23;17:1122163. doi: 10.3389/fnbeh.2023.1122163 (PMC9995972; doi:10.3389/fnbeh.2023.1122163)
Supplement: Supplementary file 7 [file Table_6.docx]

**Supplementary Table 6. C-fos immunoreactivity.**  The values shown correspond to the mean ± standard error of the number of c-Fos positive cells/area m2 in the sham, DBS, sham-FG, and FG-DBS groups. Letters indicate groups statistically different (p<0.05, Kruskal Wallis Test; Cross: p<0.08). Bold Shows differences between sexes (p<0.05, Mann-Whitney U test).

| **Brain area** | **Area** $\mu$**m2** | **Sex** | **Sham** | **DBS** | **Sham-FG** | **FG-DBS** |
| --- | --- | --- | --- | --- | --- | --- |
| **Cortical regions** |  |  |  |  |  |  |
| Cingulate cortex | 500 | Males | 43.4 ± 3.7 a | **81.2 ± 6.0 b** | **98.4 ± 4.0 b** | 51.8 ± 3.5 a |
|  | 500 | Females | 40.8 ± 6.7 | **55.2** ± **5.4** | **56.4** ± **5.6** | 50.2 ± 3.0 |
| Ectorrinal cortex | 500 | Males | 11.4 ± 2.2 a | 9.8 ± 2.5 a | **49.2 ± 7.9 b** | 10.8 ± 2.4 a |
|  | 500 | Females | 6.2 ± 1.0 | 7.2 ± 1.0 | **10.0 ± 1.4** | 7.0 ± 1.4 |
| Entorrinal cortex | 500 | Males | 15.0 ± 1.2 a | 10.0 ± 2.6 a | **45.2 ± 4.0 b** | 11.4 ± 1.4 a |
|  | 500 | Females | 13.8 ± 3.1 | 12.0 ± 2.2 | **15.4** ± **1.9** | 11.8 ± 3.7 |
| Frontal cortex | 500 | Males | 46.0 ± 3.2 a | 65.4 ± 6.4 a | 80.2 ± 6.1 b**†** | 58.2 ± 4.7 a |
|  | 500 | Females | 47.6 ± 4.1 | 56.8 ± 6.1 | 71.0 ± 4.8 | 55.4 ± 8.5 |
| Insular agranular cortex | 500 | Males | 38.8 ± 4.8 a | 72.0 ± 8.4 b | **89.6 ± 7.9 b** | 47.6 ± 3.0 a |
|  | 500 | Females | 43.0 ± 5.7 | 54.8 ± 3.5 | **49.0 ± 4.4** | 53.8 ± 8.0 |
| Infralimbic cortex | 500 | Males | 38.2 ± 5.9 a | **76.8 ± 4.8 b** | **82.6 ± 5.4 b** | 52.4 ± 3.5 a |
|  | 500 | Females | 42.6 ± 3.8 a | **59.4** ± **6.0 b** | **63.6** ± **4.3 b** | 55.0 ± 5.3 a |
| Orbital lateral cortex | 500 | Males | 45.8 ± 4.6 | 56.8 ± 6.3 | 68.2 ± 7.6 | 51.6 ± 4.0 |
|  | 500 | Females | 43.4 ± 4.1 | 57.6 ± 5.6 | 55.2 ± 10.2 | 50.6 ± 5.6 |
| Orbital ventral cortex | 500 | Males | 51.2 ± 4.5 a | 62.6 ± 6.0 a | 81.8 ± 6.5 b**†** | 56.6 ± 5.3 a |
|  | 500 | Females | 48.8 ± 7.5 | 68.6 ± 8.7 | 74.8 ± 5.9 | 54.2 ± 3.5 |
| Peduncular cortex | 500 | Males | 34.8 ± 3.5 a | 55.6 ± 7.6 b**†** | 67.8 ± 5.1 b**†** | 45.8 ± 3.5 a |
|  | 500 | Females | 40.6 ± 7.3 a | 41.6 ± 5.9 a | 73.2 ± 8.2 b | 46.8 ± 2.5 a |
| Piriform cortex | 500 | Males | 23.2 ± 2.3 a | 25.2 ± 4.8 a | 56.8 ± 4.4 b | 27.0 ± 5.5 a |
|  | 500 | Females | 21.3 ± 2.7 | 27.8 ± 3.2 | 46.6 ± 5.1 | 26.4 ± 1.7 |
| Prelimbic cortex | 500 | Males | 36.2 ± 3.2 a | 71.2 ± 5.8 b | **82.0 ± 4.1 b** | 56.4 ± 6.7 a |
|  | 500 | Females | 46.2 ± 7.5 | 45.8 ± 7.6 | **56.2** ± **6.6** | 48.0 ± 8.0 |
| Dorsal tenia tecta | 500 | Males | 64.2 ± 4.6 | 57.4 ± 5.5 | 60.6 ± 8.7 | 62.8 ± 7.1 |
|  | 500 | Females | 46.2 ± 7.5 | 41.8 ± 5.9 | 56.2 ± 6.6 | 48.0 ± 7 |
| Ventral tenia tecta | 500 | Males | **19.4 ± 2.5 a** | **22.4 ± 3.2 a** | 49.6 ± 8.4 b | **24.4 ± 3.0 a** |
|  | 500 | Females | **43.8** ± **5.2** | **45.6** ± **5.8** | 38.8 ± 8.9 | **40.8** ± **6.9** |
| Septal area |  |  |  |  |  |  |
| Lateral septum – dorsal | 500 | Males | 2.0 ± 0.7 a | 2.8 ± 0.8 b | 2.4 ± 1.2 b | 2.8 ± 1.0 a |
|  | 500 | Females | 1.4 ± 0.5 | 2.0 ± 0.9 | 1.6 ± 0.9 | 1.8 ± 1.4 |
| Lateral septum-intermediate | 500 | Males | 2.4 ± 0.8 a | 3.0 ± 0.9 a | **8.2 ± 1.3 b** | 1.6 ± 0.4 a |
|  | 500 | Females | 1.8 ± 0.9 | 2.4 ± 1.1 | **2.0** ± **0.8** | 1.4 ± 0.4 |
| Lateral septum – ventral | 500 | Males | 3.0 ± 0.6 | 2.4 ± 0.9 | 3.2 ± 1.3 | 2.8 ± 0.7 |
|  | 500 | Females | 2.6 ± 1.0 | 3.0 ± 0.8 | 2.4 ± 1.2 | 2.0 ± 0.6 |
| Medial septum | 500 | Males | 2.2 ± 0.9 a | 2.8 ± 1.0 a | **13.4 ± 1.5 b** | 3.0 ± 1.1 a |
|  | 500 | Females | 2.8 ± 1.2 | 3.0 ± 1.1 | **3.6 ± 0.9** | 2.4 ± 1.0 |
| Diagonal band of Broca | 500 | Males | 3.2 ± 0.7 a | 3.4 ± 0.5 a | **21.8 ± 2.8 b** | 3.6 ± 0.5 a |
|  | 500 | Females | 2.4 ± 0.7 | 3.4 ± 0.8 | **4.6 ± 0.7** | 3.6 ± 1.2 |
| **Hippocampus** |  |  |  |  |  |  |
| Dorsal hippocampus - CA1 | 200 | Males | 1.4 ± 0.5 a | 1.6 ± 0.4 a | **7.0 ± 1.0 b** | 1.6 ± 0.5 a |
|  | 200 | Females | 1.4 ± 0.2 | 1.3 ± 0.7 | **2.0** ± **0.3** | 1.2 ± 0.4 |
| Dorsal hippocampus – CA2 | 200 | Males | 1.0 ± 0.3 | 1.2 ± 0.2 | 1.2 ± 0.6 | 1.0 ± 0.4 |
|  | 200 | Females | 1.2 ± 0.4 | 0.8 ± 0.2 | 1.0 ± 0.3 | 1.4 ± 0.5 |
| Dorsal hippocampus – CA3 | 200 | Males | 1.6 ± 0.4 | 1.6 ± 0.2 | 2.6 ± 0.7 | **2.0 ± 0.3** |
|  | 200 | Females | 0.8 ± 0.4 | 0.8 ± 0.5 | 1.0 ± 0.4 | **0.6** ± **0.4** |
| Dorsal hippocampus – Dentate gyrus | 200 | Males | 1.4 ± 0.2 | 1.6 ± 0.2 | **2.8 ± 0.7** | **1.6 ± 0.4** |
|  | 200 | Females | 1.0 ± 0.5 | 1.0 ± 0.3 | **0.8** ± **0.4** | **0.4** ± **0.2** |
| Intermediate hippocampus - CA1 | 200 | Males | 2.4 ± 0.5 | 2.8 ± 0.7 | 2.6 ± 0.9 | 2.7 ± 0.7 |
|  | 200 | Females | 1.6 ± 0.5 | 1.2 ± 0.4 | 1.8 ± 0.7 | 2.0 ± 0.5 |
| Ventral hippocampus - CA1 | 200 | Males | 2.0 ± 0.4 a | 2.2 ± 1.0 a | **7.4 ± 1.4 b** | 1.6 ± 0.5 a |
|  | 200 | Females | 2.2 ± 0.4 | 2.0 ± 0.4 | **2.4** ± **0.5** | 1.8 ± 0.6 |
| Ventral hippocampus – CA2 | 200 | Males | 1.8 ± 0.4 | **1.8 ± 0.4** | 2.0 ± 0.7 | **2.2 ± 0.5** |
|  | 200 | Females | 1.0 ± 0.3 | **0.6** ± **0.2** | 0.8 ± 0.4 | **0.4** ± **0.2** |
| Ventral hippocampus – CA3 | 200 | Males | 1.4 ± 0.5 | 1.7 ± 0.4 | 1.4 ± 0.6 | 1.6 ± 0.6 |
|  | 200 | Females | 1.4 ± 0.5 | 1.6 ± 0.4 | 19.0 ± 2.0 | 1.6 ± 0.5 |
| Ventral hippocampus - dentate gyrus | 200 | Males | 3.0 ± 0.7 | 3.4 ± 0.7 | 3.1 ± 1.1 | 2.4 ± 0.7 |
|  | 200 | Females | 1.4 ± 0.5 | 1.9 ± 0.7 | 1.6 ± 0.7 | 1.8 ± 0.6 |
| **Amygala** |  |  |  |  |  |  |
| Basolateral amygdala | 200 | Males | 3.2 ± 0.7 a | 2.8 ± 0.9 a | **16.2 ± 1.8 b** | 4.0 ± 0.7 a |
|  | 200 | Females | 3.0 ± 0.3 | 2.7 ± 0.6 | **4.4 ± 0.9** | 3.8 ± 0.6 |
| Basomedial amygdala | 200 | Males | **3.0 ± 0.7** | 2.4 ± 1.1 | **3.4 ± 0.7** | 2.6 ± 0.9 |
|  | 200 | Females | **1.0** ± **0.3** | 0.8 ± 0.2 | **1.2** ± **0.4** | 1.4 ± 0.5 |
| Central amygdala | 200 | Males | 7.2 ± 1.0 a | 6.8 ± 1.3 a | **22.0 ± 2.5 b** | 8.2 ± 1.1 a |
|  | 200 | Females | 5.6 ± 0.6 | 5.0 ± 0.8 | **6.4** ± **0.7** | 4.6 ± 0.8 |
| Lateral amygdala | 200 | Males | 2.4 ± 0.4 | 2.0 ± 0.7 | 2.6 ± 0.8 | 1.8 ± 0.4 |
|  | 200 | Females | 1.0 ± 0.4 | 0.8 ± 0.4 | 1.2 ± 0.4 | 1.0 ± 0.5 |
| Medial amygdala | 200 | Males | **4.0 ± 0.7** | **4.0 ± 0.7** | **11.4 ± 1.0** | **3.4 ± 1.0** |
|  | 200 | Females | **2.2** ± **0.6** | **2.2** ± **0.4** | **2.0** ± **0.3** | **2.0** ± **0.5** |
| Amygdalo-piriform transition zone | 200 | Males | 3.6 ± 0.7 a | 4.2 ± 0.6 a | **13.2 ± 2.2 b** | 5.2 ± 1.2 a |
|  | 200 | Females | 5.0 ± 0.7 | 4.4 ± 0.5 | **7.6 ± 1.1** | 3.6 ± 2.5 |
| Posterolateral cortical amygdaloid nucleus | 200 | Males | **6.6 ± 0.7** | **7.4 ± 0.5** | **8.0 ± 2.4** | **9.2 ± 1.1** |
|  | 200 | Females | **3.2** ± **0.7** | **3.0** ± **0.6** | **2.4** ± **0.7** | **4.0** ± **0.9** |
| Posteromedial cortical amygdaloid nucleus | 200 | Males | 8.8 ± 1.8 a | 7.8 ± 1.8 a | **18.4 ± 2.3 b** | 10.2 ± 0.7 a |
|  | 200 | Females | 5.8 ± 0.6 | 7.2 ± 0.8 | **9.0** ± **1.5** | 6.4 ± 0.7 |
| Amygdalo-hippocampal área | 200 | Males | **8.0 ± 1.2** | 6.2 ± 1.2 | 7.4 ± 2.7 | **9.0** ± **1.2** |
|  | 200 | Females | **4.8** ± **0.7** | 5.2 ± 0.6 | 6.8 ± 1.3 | **4.6** ± **0.7** |
| Endopiriform nucleus | 200 | Males | 14.8 ± 2.2 | 16.2 ± 1.7 | 26.0 ± 2.6 | 17.4 ± 2.4 |
|  | 200 | Females | 10.8 ± 1.4 | 9.6 ± 2.1 | 16.2 ± 1.7 | 11.4 ± 1.8 |
| **Diencephalon** |  |  |  |  |  |  |
| Centromedian thalamic nucleus | 200 | Males | 12.6 ± 1.2 | 11.8 ± 1.0 | 17.8 ± 3.3 | 9.8 ± 1.6 |
|  | 200 | Females | 9.2 ± 2.0 | 11.4 ± 3.0 | 13.6 ± 2.4 | 10.6 ± 1.2 |
| Paraventricular thalamic nucleus | 200 | Males | 5.6 ± 1.7 a | 7.8 ± 1.2 a | 15.2 ± 1.4 b | 6.8 ± 1.3 a |
|  | 200 | Females | 8.4 ± 1.3 a | 11.2 ± 1.7 a | 18.8 ± 1.4 b | 11.4 ± 1.9 a |
| Reuniens nucleus | 200 | Males | 9.6 ± 1.2 | 8.2 ± 0.9 | 10.6 ± 2.4 | 7.4 ± 0.7 |
|  | 200 | Females | 8.0 ± 1.0 | 7.6 ± 1.6 | 8.4 ± 1.0 | 10.7 ± 1.3 |
| Rhomboidal nucleus | 200 | Males | 12.4 ± 1.2 | 11.4 ± 1.6 | 13.2 ± 4.0 | 14.8 ± 2.9 |
|  | 200 | Females | 11.2 ± 1.8 | 9.4 ± 1.6 | 10.0 ± 1.7 | 10.6 ± 1.2 |
| Paraventricular hipothalamic nucleus | 200 | Males | 9.0 ± 1.2 a | 15.2 ± 2.2 a | 42.6 ± 4.0 b | 19.8 ± 3.8 a |
|  | 200 | Females | 13.4 ± 2.9 a | 26.6 ± 5.1 a | 50.4 ± 4.7 b | 21.4 ± 2.7 a |
| **Brainstem** |  |  |  |  |  |  |
| Dorsal Raphe | 200 | Males | 2.4 ± 0.5 a | 2.6 ± 0.7 a | **18.0 ± 1.4 b** | 3.2 ± 0.6 a |
|  | 200 | Females | 1.8 ± 0.4 a | 4.0 ± 1.4 a | **10.2** ± **0.8 b** | 2.2 ± 0.6 a |
| Locus coeruleus | 200 | Males | 7.6 ± 1.1 a | 8.2 ± 2.1 a | 18.2 ± 3.4 b | **9.0 ± 1.1 a** |
|  | 200 | Females | 6.0 ± 0.9 a | 6.4 ± 1.3 a | 16.8 ± 2.5 b a | **4.4** ± **1.4 a** |
| Nucleus incertus | 200 | Males | 2.0 ± 0.71 a | 2.6 ± 0.8 a | **15.8 ± 1.7 b** | 3.0 ± 0.7 a |
|  | 200 | Females | 2.6 ± 0.7 | 2.4 ± 0.9 | **2.6** ± **1.3** | 2.4 ± 1.0 |
| Lateral parabraquial nucleus | 200 | Males | 4.4 ± 0.7 | 6.2 ± 2.4 | 5.8 ± 0.7 | 6.4 ± 0.8 |
|  | 200 | Females | 3.8 ± 0.4 | 4.2 ± 0.9 | 5.0 ± 1.4 | 4.8 ± 1.5 |
| Periaqueductal grey substance | 200 | Males | 2.0 ± 0.3 | 3.4 ± 0.9 | 6.8 ± 1.9 | 2.8 ± 0.7 |
|  | 200 | Females | 3.4 ± 0.8 | 3.8 ± 1.0 | 5.2 ± 0.9 | 4.4 ± 0.6 |

Mean ± standard error. **Bold**: statistical significance between sexes; asterisks denote statistical significance between states ***p<0.001, **p<0.01, *p<0.05. BLA: basolateral amygdala; HPCd: dorsal hippocampus; HPCi: intermediate hippocampus; HPCv: ventral hippocampus.

±
